# Chitosan-Modified Polyethyleneimine Nanoparticles for Enhancing the Carboxylation Reaction and Plants’ CO_2_ Uptake

**DOI:** 10.1021/acsnano.2c09255

**Published:** 2023-02-16

**Authors:** Cyril Routier, Lorenzo Vallan, Yohann Daguerre, Marta Juvany, Emin Istif, Daniele Mantione, Cyril Brochon, Georges Hadziioannou, Åsa Strand, Torgny Näsholm, Eric Cloutet, Eleni Pavlopoulou, Eleni Stavrinidou

**Affiliations:** † Laboratory of Organic Electronics, Department of Science and Technology, Linköping University, SE-60174 Norrköping, Sweden; ‡ Laboratoire de Chimie des Polymères Organiques (LCPO−UMR 5629), Université de Bordeaux, Bordeaux INP, CNRS, F-33607 Pessac, France; § Umeå Plant Science Centre, Department of Forest Genetics and Plant Physiology, Swedish University of Agricultural Sciences, SE-90183 Umeå, Sweden; ⊥ POLYMAT, University of the Basque Country UPV/EHU, 20018 San Sebastián, Spain; ¶ Umeå Plant Science Centre, Department of Plant Physiology, Umeå University, SE 901-87 Umeå, Sweden; # Institute of Electronic Structure and Laser, Foundation for Research and Technology—Hellas, P.O. Box 1527, 71110 Heraklion Crete, Greece

**Keywords:** nanoparticles, CO_2_ capture, polyethyleneimine, chitosan, photosynthesis

## Abstract

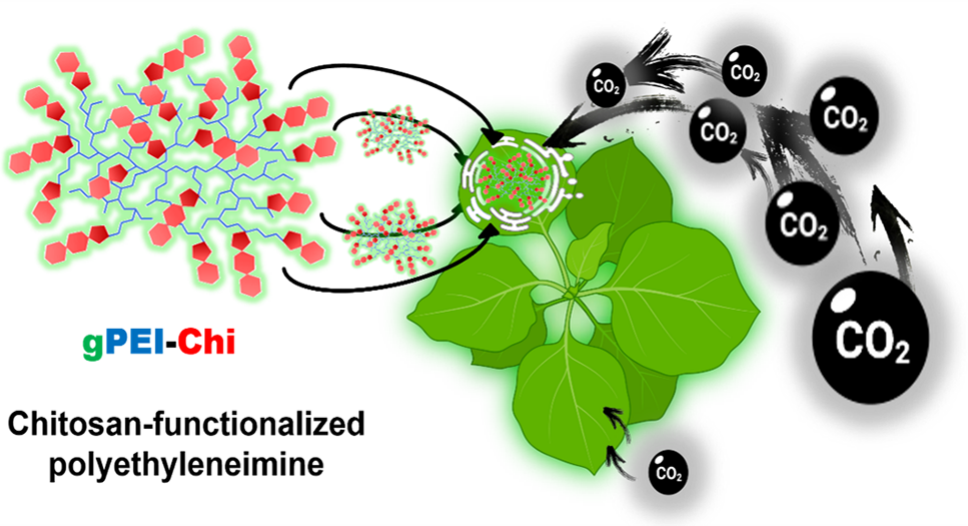

Increasing plants’ photosynthetic efficiency is a major challenge that must be addressed in order to cover the food demands of the growing population in the changing climate. Photosynthesis is greatly limited at the initial carboxylation reaction, where CO_2_ is converted to the organic acid 3-PGA, catalyzed by the RuBisCO enzyme. RuBisCO has poor affinity for CO_2_, but also the CO_2_ concentration at the RuBisCO site is limited by the diffusion of atmospheric CO_2_ through the various leaf compartments to the reaction site. Beyond genetic engineering, nanotechnology can offer a materials-based approach for enhancing photosynthesis, and yet, it has mostly been explored for the light-dependent reactions. In this work, we developed polyethyleneimine-based nanoparticles for enhancing the carboxylation reaction. We demonstrate that the nanoparticles can capture CO_2_ in the form of bicarbonate and increase the CO_2_ that reacts with the RuBisCO enzyme, enhancing the 3-PGA production in *in vitro* assays by 20%. The nanoparticles can be introduced to the plant *via* leaf infiltration and, because of the functionalization with chitosan oligomers, they do not induce any toxic effect to the plant. In the leaves, the nanoparticles localize in the apoplastic space but also spontaneously reach the chloroplasts where photosynthetic activity takes place. Their CO_2_ loading-dependent fluorescence verifies that, *in vivo*, they maintain their ability to capture CO_2_ and can be therefore reloaded with atmospheric CO_2_ while *in planta*. Our results contribute to the development of a nanomaterials-based CO_2_-concentrating mechanism in plants that can potentially increase photosynthetic efficiency and overall plants’ CO_2_ storage.

During photosynthesis, plants capture carbon dioxide (CO_2_) and, by utilizing light energy, convert it to carbohydrates, which is the energy source of most heterotrophic organisms but also a source of fibers and biofuels. With the growing population, saturation of resources, and the climate crisis, there is a great need to improve plants photosynthetic efficiency and increase crop yield. One of the major limiting steps of photosynthesis occurs during the initial carboxylation reaction for carbon fixation in the chloroplasts. During this step of the Calvin cycle, CO_2_ is converted to the organic acid 3-PGA (3-phosphoglycerate) catalyzed by the RuBisCO (Ribulose 1,5-bisphosphate carboxylase/oxygenase) enzyme.^1,2^ While RuBisCO has naturally poor affinity for CO_2_, its dual nature as oxygenase also leads to a competing reaction with O_2_, creating the wasteful pathway of photorespiration that reduces the Calvin cycle’s efficiency by decreasing the photosynthetic carbon fixation of more than 25%.^3,4^ Furthermore, to reach the RuBisCO enzyme, atmospheric CO_2_ has to follow a resistive pathway and diffuse through the leaf stomata, the intercellular space, the cell membrane, cytoplasm, and finally the chloroplast envelopes.^5,6^ Thus, in order to sustain their growth, plants synthesize a large number of RuBisCO enzymes, which requires high nitrogen use, a process that can be problematic for the plant development when nitrogen availability is limited.

Despite great progress in understanding RuBisCO biosynthesis and catalytic action, but also advances on plastid transformation^7^ and RuBisCO recombinant expression,^8,9^ improving RuBisCO’s catalytic activity and specificity to CO_2_ in crop plants via genetic engineering remains a complex and challenging endeavor.^10,11^ Apart from direct enzyme engineering, another promising route is to increase the amount of CO_2_ in the vicinity of RuBisCO via a CO_2_-concentrating mechanism,^12^ either biochemically, such as C4 photosynthesis^13,14^ or through the expression of inorganic carbon transporters such as those for bicarbonate.^15^

Beyond genetic engineering, other approaches to enhance or augment plant functions based on materials science and nanotechnology have emerged.^16−18^ Synthetic materials can be rationally designed with desired properties that are optimized independently of the biological environment. However, it is challenging to introduce the synthetic materials into the biological organism in a seamless manner and incorporate the synthetic functionality into the biological machinery. In the literature, several examples at the proof-of-concept level exist. Functionalized nanotubes have been introduced to plants for *in vivo* detection of endogenous analytes such as H_2_O_2_^19^ or to convert plants to environmental sensors for detecting exogenous analytes such as arsenic.^20^ Quantum dots have been used for *in vivo* targeted delivery of biochemicals,^21^ while conjugated polymer nanoparticles were used for controlling plant functions via light stimulation.^22^ Our group, on the other hand, focused on the development of plant biohybrids using the biochemistry of plants to polymerize *in vivo* conducting polymers that can be used for energy storage.^23−25^

Enhancing photosynthesis with synthetic materials has been attempted by mostly focusing on improving the light-dependent reactions. Semiconducting nanoparticles that localized in the proximity of the thylakoids have shown to improve the electron transport rates^26,27^ and/or act as antennas, extending the light absorption spectrum of the plant.^28,29^ To date, leveraging nanomaterials for enhancing the CO_2_ uptake of plants has not been demonstrated.

Polyethyleneimine-based materials are promising candidates as they have an excellent CO_2_ capture ability as it was demonstrated by several studies focusing on the design of new CO_2_ absorbents for postcombustion processes.^30−32^ The highly dense amines of polyethyleneimine (PEI) react with CO_2_, forming zwitterionic species such as carbamic acids and carbamates or, in the presence of water, the corresponding ammonium hydrogen carbonate salts. Furthermore, PEI or PEI-coated nanomaterials can cross plant cellular barriers and localize in the cytosol or organelles, as it was demonstrated both *in vitro*(33,34) and *in vivo* studies. Particularly, PEI has been used as carrier to deliver plasmids to plants for transient gene expression,^35^ as well as to deliver small interfering RNA, siRNA, for gene silencing.^36^ PEI’s high positive charge condensates negatively charged polynucleotides and, at the same time, protects them from degradation, thus improving transfection efficiency. Yasumoto et al. demonstrated that small biogenic polyamines, such as piperazine and putrescine, can capture atmospheric CO_2_ which can then be used as a substrate for the carboxylation reaction.^37^ However, they have not studied their introduction to plants or their phytotoxicity. Other polyamines, such as PEI, can be highly toxic to plants depending on their molecular structure and concentration used.^38^

In this work, we designed phytocompatible green-fluorescent PEI-based nanoparticles that can capture and transfer CO_2_ to RuBisCO for the carboxylation reaction. The phytotoxicity of the nanoparticles is inhibited by the presence of protective chitosan oligomers, while the fluorophore functionalization enables CO_2_ loading-dependent fluorescence that does not overlap with the leaf autofluorescence. The nanoparticles were introduced to the plant via infiltration, and they spontaneously localized within the chloroplasts where photosynthesis takes place.

## Results and Discussion

**Figure 1:**
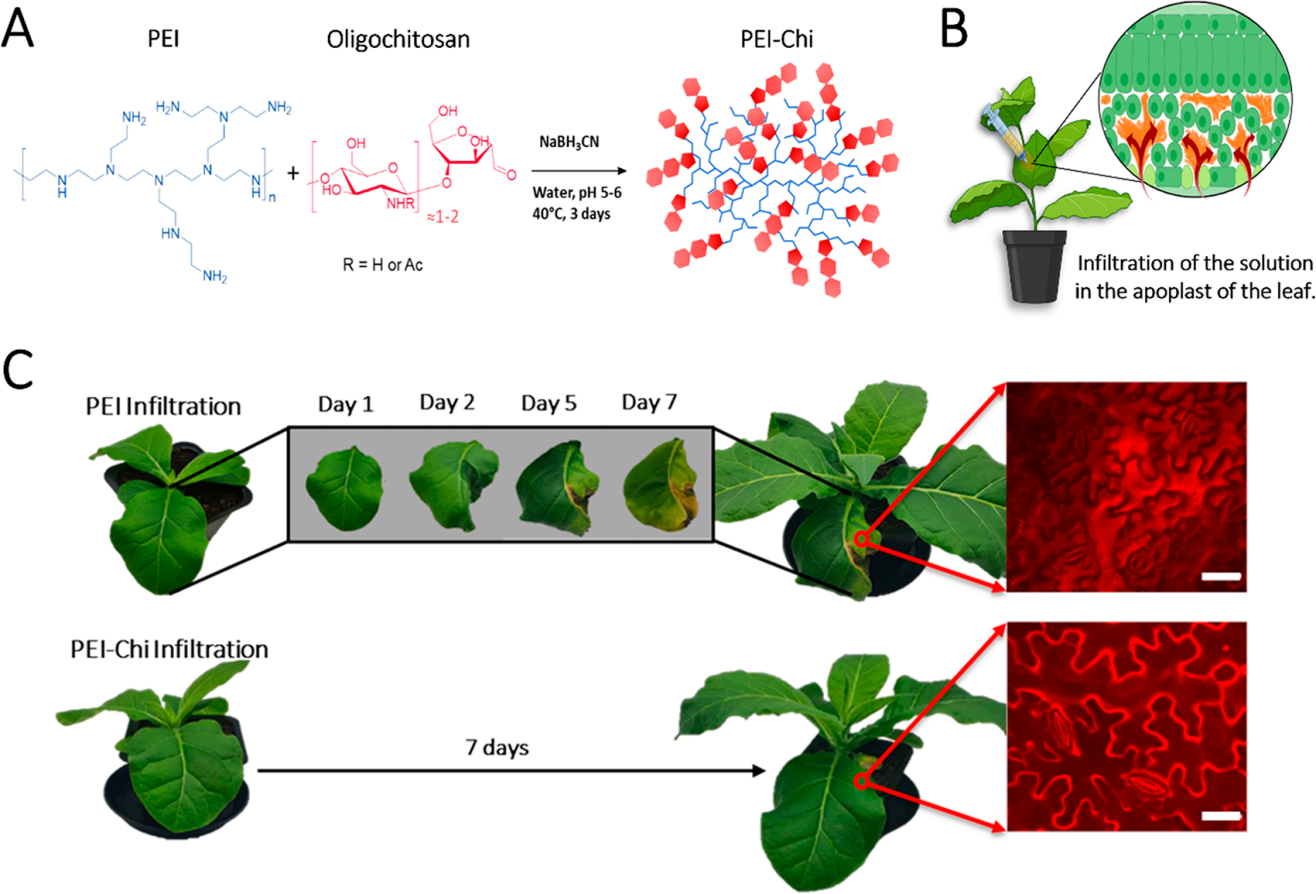
Infiltration of PEI-Chi in *Nicotiana tabacum* (tobacco) plants and evaluation of its phytotoxicity (A) Functionalization of **PEI** with oligochitosan by reductive amination. (B) Scheme of the infiltration method: a solution contained in a needleless syringe is infiltrated in the abaxial part of a leaf by gently applying pressure. The solution is pushed in the intercellular space existing between the cells’ membrane called apoplast (created with BioRender.com). (C) Comparison of the effects of the infiltration of **PEI** (above) and **PEI-Chi** (below) on the tobacco leaf. **PEI** infiltration leads to a quick damage in the infiltrated area and around it while the infiltration of **PEI-Chi** does not show any visible sign of toxicity even one month after infiltration (Figure S14). The insets on the right show fluorescent imaging obtained after staining of the samples with propidium iodide, a cationic dye that does not cross intact membranes but binds to cell walls, forming an outline of living cells. Scale bar: 50 μm.

Branched PEI of 2 kDa molecular weight was chosen as an initial potential candidate for enhancing plants’ CO_2_ uptake and carbon fixation. However, infiltration of branched PEI in *Nicotiana tabacum* (tobacco) leaves revealed its toxicity for the plant as it will be discussed in detail in the next section. The synthesis of polymers containing both ethylenamide and glycoside units recently proved to be a promising strategy for the preparation of nontoxic gene vectors.^39−41^ Inspired by this approach, we modified 2 kDa PEI with chitosan oligomers. Chitosan is obtained from the partial deacetylation of chitin, the constituent of several crustaceans and insects exoskeleton, and therefore, it is the second most abundant polymer in nature after cellulose. As an emerging biocompatible, biodegradable, and cheap material, chitosan and chitosan nanoparticles have found use in several fields, such as packaging,^42^ gene delivery,^43^ enzyme immobilization,^44^ water treatment,^45^ and agriculture.^46^ Chitosan-based materials have been used in plant applications for enhancing seedling growth,^47^ controlled release of nutrients,^48^ and for antifungal and antibacterial purposes.^49^ First, chitosan oligomers (oligochitosan), of average 2–3 sugar units (Figure S1), were prepared by nitrous acid-promoted depolymerization of chitosan (MW 15 kDa), exploiting a previously reported procedure that allows to roughly control the size of the oligomers through the stoichiometry of the reaction.^50^ In contrast with the chitosan polymer, these oligosaccharides are highly soluble in water even at neutral pH. Moreover, the cleavage of the glycosidic linkage during the depolymerization reaction forms a 2,5-anhydro-d-mannofuranose reducing-end, whose aldehyde group does not participate in intramolecular hemiacetals and can be exploited for further reactions. In the second step, we performed a reductive amination reaction between the amines of PEI and the aldehydes of oligochitosans. By forming a C–N covalent bond between PEI and oligochitosan, this synthetic route converts the primary amines of PEI to secondary amines and the secondary amines to tertiary amines. In this way, PEI-Chi was obtained, consisting of a PEI branched molecule covered by numerous oligochitosan functionalities (Figure 1A). It should be noted that this functionalization strategy does not consume PEI amine groups, thus not reducing the polymer’s ability to capture CO_2_. **PEI-Chi** structure was investigated by nuclear magnetic resonance (NMR) spectroscopy. In the proton (^1^H) and carbon (^13^C) APT (attached proton test) NMR spectra of **PEI-Chi**, the signals of the oligochitosan and PEI parts are present, whereas the aldehyde signal, located at 5.01 ppm in the ^1^H spectrum of oligochitosan, has disappeared in the spectrum of **PEI-Chi**, demonstrating that the functionalization has taken place (Figures S2–S6). Finally, we labeled **PEI-Chi** with a small amount of fluorescein isothiocyanate (FITC), whose fluorescence can be exploited for *in vivo* localization studies. By washing with ethanol and acetone, the free dye was completely removed, and we isolated the green-fluorescent **PEI-Chi (gPEI-Chi)**. The NMR spectra of **gPEI-Chi** showed no significant differences from those of **PEI-Chi**, except for weak proton signals between 7.7 and 6.5 ppm, which suggest the presence of bound FITC (Figures S7–S9). Infrared spectroscopy (IR) analysis of oligochitosan, **PEI-Chi** and **gPEI-Chi** showed the typical vibrational features of polysaccharides (Figure S10). In detail, the spectrum of gPEI-Chi shows wide and multiple absorption bands between 3600 and 3300 cm^–1^, which correspond to O–H and N–H stretching, free or involved in hydrogen bonds. Between 2928 and 2868 cm^–1^ it is observed the C–H symmetric and asymmetric stretching of the glycans and PEI skeleton, while the bands at 1649 and 1560 cm^–1^ correspond to amide I and amide II vibrational modes. Even N–H bending probably contributes to the intensity of those bands, as features in the same area are present in the spectrum of bare PEI. The signals between 1414 and 1317 cm^–1^ were attributed to O–H bending, while C–OH and C–O–C stretching mainly contributes to the peaks located at 1149, 1069, and 1024 cm^–1^. An additional contribution to the absorption in this range is given by the C–N stretching, which also appears in the spectrum of bare PEI at 1117 and 1042 cm^–1^.^51^ Atomic force microscopy (AFM) characterization of **gPEI-Chi** (Figure S11) showed that the sample is composed of individual nanoparticles with 2.0–2.5 nm size and of larger particles, which could consist of aggregates of about 5 nm. For comparison, the individual nanoparticles of bare PEI show an average size of 1.0–1.5 nm, therefore the larger size of **gPEI-Chi** is due to the oligochitosan functionalization. Size exclusion chromatography (SEC) also confirmed the increase of size, estimating a molecular weight of **gPEI-Chi** more than doubled with respect to bare PEI (Figure S12 and Table S13). In addition, the dispersity of **gPEI-Chi** is higher than that of bare PEI as a result of the functionalization.

Next, we proceeded to characterize the phytotoxicity of **gPEI-Chi** and compared it with bare branched PEI. Both polymers were infiltrated in the apoplast of 6 weeks old *Nicotiana tabacum* (tobacco) leaves (Figure 1B). To maintain cell homeostasis, the materials were dissolved in a typical aqueous buffer used for infiltration, consisting of 2-(N-morpholino)ethanesulfonic acid (MES) (pH 5.6) and MgCl_2_, both in the concentration of 10 mM.^52−54^ The plants were visually inspected for a minimum of 7 days after infiltration. As shown in Figure 1C, the leaves infiltrated with bare PEI stopped growing with clear toxicity effects. The infiltrated area dried out and became brittle, while the adjacent tissue exhibited chlorosis. Indeed, it has been found that PEI in PEI-functionalized nanotubes induces transcriptional reprogramming in plants, leading to stress, immune responses, and senescence that may lead to cell death and tissue damage depending on the concentration of PEI and structure.^55^

In contrast, the plants infiltrated with **gPEI-Chi** did not show any visible sign of toxicity for more than a week and the infiltrated leaves continued to grow normally. To verify the tissue integrity of the infiltrated leaves, we performed histochemical staining with Propidium Iodide (PI). PI is a fluorescent dye that binds to the plant cell wall and cannot cross the intact cell membrane (insets in Figure 1C). The area of leaves infiltrated with PEI could not be stained with PI as it dried out with no intact cells for efficient staining. The adjacent discolored tissue was stained with the dye entering the cells, indicating that the membranes were damaged and the cells dying. Instead, in the case of **gPEI-Chi** infiltrated leaves, the PI did not cross the cell membranes, not even very close to the infiltration point, indicating that the cells are intact and that the oligochitosan functionalization effectively improved the phytocompatibility of PEI.

**Figure 2:**
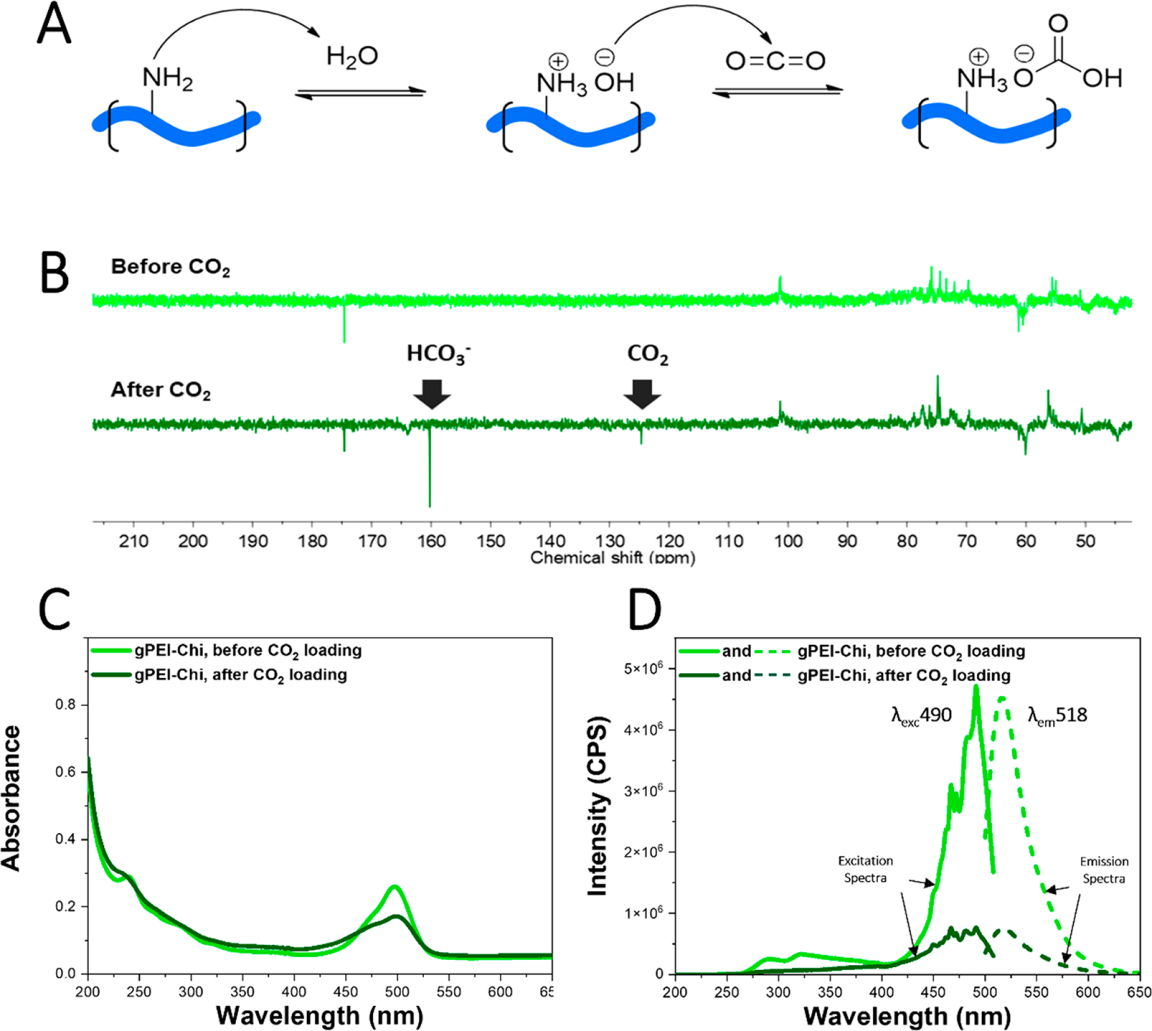
Functionalization of **PEI-Chi** for fluorescent detection *in vivo* and CO_2_ uptake emission dependence. (A) Scheme of the ammonium bicarbonate formation from the reaction between amine and CO_2_ in aqueous environment (PEI contains primary, secondary, and tertiary amines and all three kinds can react with CO_2_. We here used primary amines only as a showcase). (B) APT NMR spectra of **PEI-Chi** in D_2_O before and after bubbling carbon dioxide. After bubbling, strong peaks corresponding to hydrogen carbonate and CO_2_ appear. (C) Absorption and (D) excitation and emission spectra of **gPEI-Chi** (0.05 mg/mL) in water before and after saturating the solution with carbon dioxide.

The ability of **PEI-Chi** to bind carbon dioxide and store it in the form of hydrogen carbonate ions was studied by NMR spectroscopy (Figure 2A,B). CO_2_ was bubbled in a deuterium oxide solution of **PEI-Chi** (30 mg/mL) and the carbon APT spectrum was recorded. A strong peak appears at 160 ppm after CO_2_ bubbling, corresponding to the HCO_3_^–^ anion. A smaller peak at 125 ppm corresponds instead to carbon dioxide, which is in equilibrium with the hydrogen carbonate. The pH of the solution shifted from 8 to 9 to 5–6 after CO_2_ bubbling, reflecting the conversion of amines into ammonium hydrogen carbonate groups. The APT experiment was repeated in absence of **PEI-Chi** under identical experimental conditions, finding that in pure D_2_O no signal of bicarbonate appeared after CO_2_ bubbling (Figure S15), thus demonstrating the essential role of **PEI-Chi** in storing CO_2_ as bicarbonate.

Then we investigated the optical properties of **gPEI-Chi** with UV–vis absorption and fluorescence emission spectroscopy (Figure 2C,D). Fluorescence emission with a maximum at 518 nm was observed for λ_exc_ = 490 nm, corresponding to the FITC fluorescence. This emission wavelength is particularly convenient for *in vivo* detection and localization studies as it does not interfere with the plant tissue autofluorescence.

Next, we studied the change in fluorescence due to the CO_2_ loading in solution. Interestingly, both absorption and fluorescence decreased significantly after CO_2_ loading. We hypothesize that prior to CO_2_ bubbling, fluorescein is present as a dianion where both the carboxylic acid and the phenol group are deprotonated due to the strong basic environment generated by the abundant surrounding amines of the **gPEI-Chi** structure. When **gPEI-Chi** reacts with carbon dioxide, the pH of the solution decreases and the phenol is restored, causing a loss of emission intensity since the monoanion form has a lower quantum yield compared to the dianion form.^56^

The decrease in fluorescence intensity due to CO_2_ loading was observable even when we used the infiltration buffer as the solvent, indicating that even local pH changes induce a change in the nanoparticles’ fluorescence (Figure S16). The CO_2_-dependent fluorescence of **gPEI-Chi** is very promising for *in vivo* studies as it may enable monitoring CO_2_ concentration dynamics, providing insight on the CO_2_ uptake and transport within the leaf.

**Figure 3:**
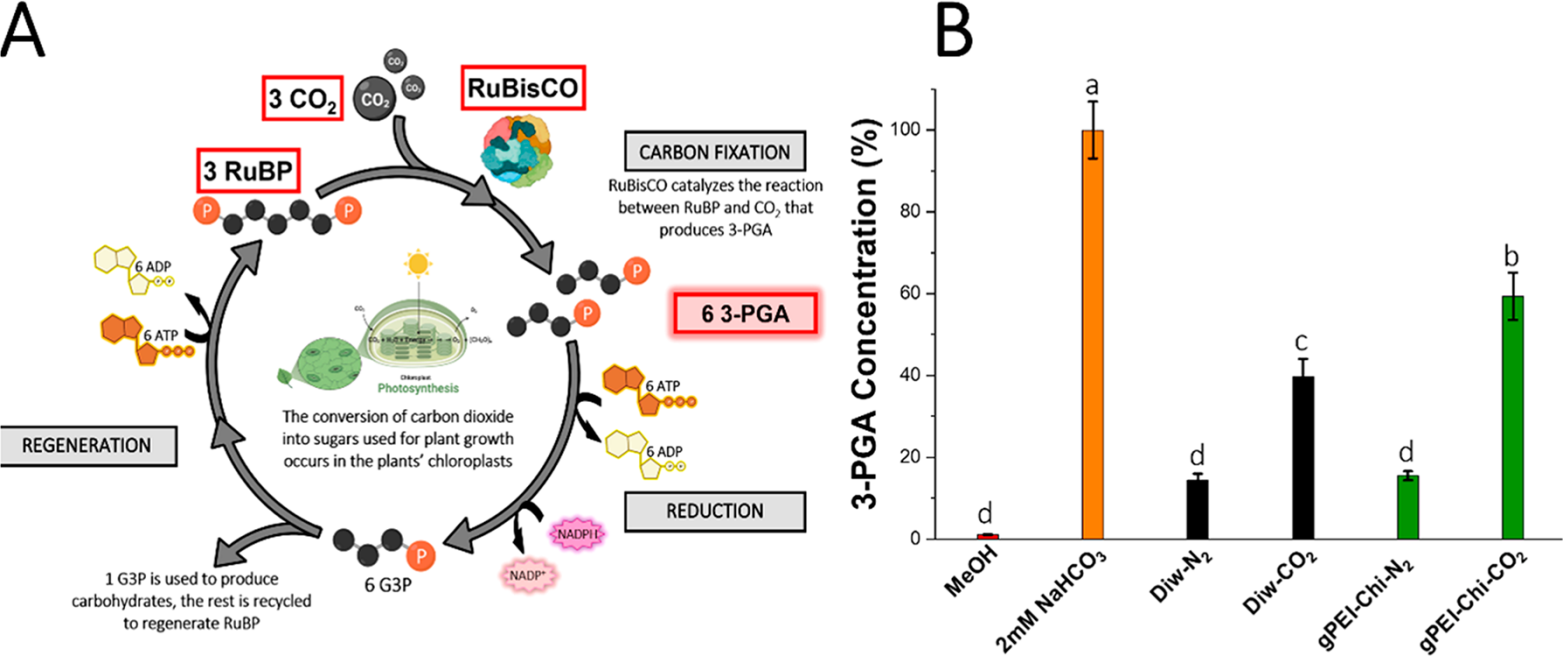
Evaluation of 3-phosphoglycerate production using **gPEI-Chi** as a CO_2_-giver to activate RuBisCO. (A) Simplified Calvin cycle showing the importance of RuBisCO as catalyst for the conversion of RuBP to 3-PGA using CO_2_ (created with BioRender.com). (B) Either CO_2_ gas or N_2_ gas, to remove CO_2_, was bubbled for 30 min in 10 mL of either 1 mg/mL of **gPEI-Chi** or distilled water. The solutions loaded with CO_2_ were then used as substrates for the carboxylation reaction between RuBP and CO_2_ catalyzed by RuBisCO and compared to their equivalent without CO_2_ treatment and to NaHCO_3_ (2 mM) as a carbon source for positive control. The amount of 3-PGA produced by the reaction was then analyzed by LC-MS. Bars indicate the standard errors (*n* = 3). Treatments not labeled by the same letter are significantly different. Tukey-Kramer HSD, *P*-value < 0.001. JMP Pro software was used to run the analysis.

After validating the ability of **gPEI-Chi** to uptake CO_2_, we proceeded to investigate whether the loaded CO_2_ can participate in biochemical reactions and specifically the carboxylation reaction. During this first phase of the Calvin cycle, CO_2_ reacts with the RuBisCO, which converts ribulose-1,5-bisphosphate (RuBP) into 3-phosphoglycerate (3-PGA) (Figure 3A).^57^

CO_2_ is electrostatically bound to **PEI-Chi** in the form of hydrogen carbonate which cannot react directly with RuBisCO. However, CO_2_ and hydrogen carbonate exist in thermodynamic equilibrium, and they dynamically interchange through reaction with water. We hypothesize that **PEI-Chi** will increase the hydrogen bicarbonate concentration and consequently the amount of CO_2_ available for RuBisCO. The ability of RuBisCO to use the CO_2_ delivered by **PEI-Chi** as substrate for the synthesis of 3-PGA *in vitro* was evaluated with an enzymatic assay adapting the method previously described by Yasumoto *et al*.^37^ We evaluated the activity of RuBisCO in five different aqueous solutions: **gPEI-Chi** in water (1 mg/mL) bubbled with N_2_ (**gPEI-Chi-N**_**2**_) or CO_2_ (**gPEI-Chi-CO**_**2**_), deionized water (DIw) bubbled with N_2_ (**DIw-N**_**2**_), or CO_2_ (**DIw-CO**_**2**_) as negative controls, and 2 mM NaHCO_3_ as positive control.

These solutions were added in a buffered solution with RuBisCO and were let to incubate for 10 min at room temperature in order to activate the enzyme. Next, RuBP was introduced, triggering the RuBisCO/CO_2_ catalyzed synthesis of 3-PGA. After 6 min, the reaction was stopped by addition of formic acid. Finally, the 3-PGA concentration in each solution was quantified with liquid chromatography-mass spectrometry (LC-MS) and normalized so that the amount of 3-PGA produced by the positive control NaHCO_3_ correspond to 100%. As shown in Figure 3B, the concentration of 3-PGA was 45% higher in the **gPEI-Chi-CO**_**2**_ solution compared to the **gPEI-Chi-N**_**2**_. Furthermore, the 3-PGA concentration in the **gPEI-Chi-CO**_**2**_ solution was 20% higher than in **DIw-CO**_**2**_, verifying that **gPEI-Chi-CO**_**2**_ can effectively increase the CO_2_ amount that reacts with RuBisCO via the hydrogen carbonate −CO_2_ conversion in water. When 2 mM NaHCO_3_ was used, the concentration of 3-PGA produced by RuBisCO was higher than that produced in the presence of **gPEI-Chi**, probably due to different concentration of captured CO_2_. However, in contrast to **gPEI-Chi**, NaHCO_3_ cannot be infiltrated in plants as it is highly toxic. Thus, these results suggest that **gPEI-Chi** could potentially improve the CO_2_ conversion of the plant by increasing the CO_2_ concentration in the RuBisCO environment.

**Figure 4:**
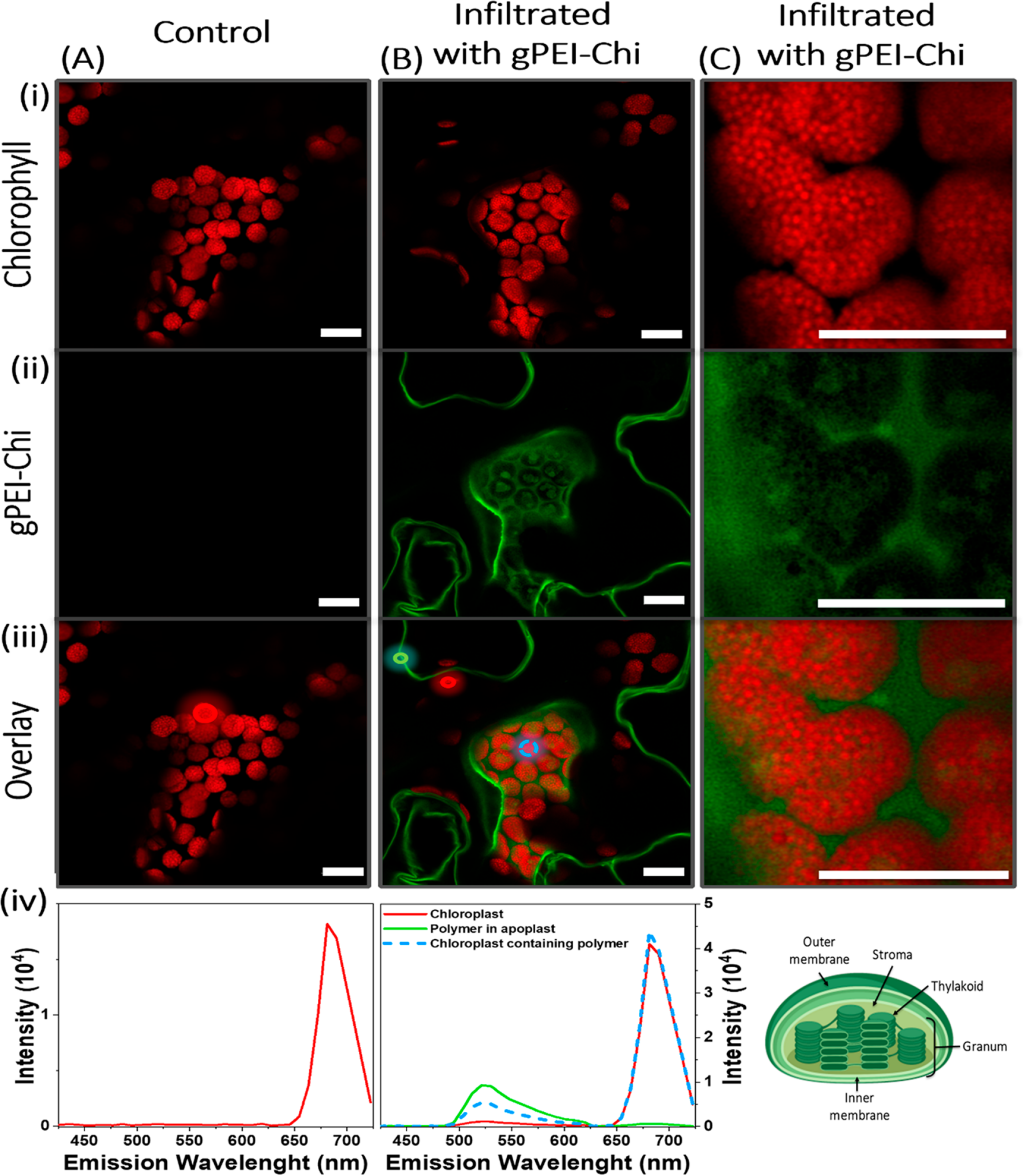
Confocal microscopy imaging to evaluate the chloroplast accessibility to **gPEI-Chi-CO_2_**. Imaging of column (A) a control tobacco leaf, column (B) and (C) a leaf infiltrated with **gPEI-Chi-CO**_**2**_. The top red panel (i) shows the autofluorescence of the chlorophyll, the middle green panel (ii) shows the **gPEI-Chi-CO**_**2**_ fluorescence, and the panel (iii) is a merging of the two previous. The graphs (A-iv) and (B-iv) are emission spectra of the circles in the merged images, obtained with a monochromator, and serve to evaluate the spatial distribution of the **gPEI-Chi-CO**_**2**_ nanoparticles in respect to the chlorophyll. The schematic (C-iv) represents the structure of a chloroplasts containing thylakoids forming stacks of disks (grana), which are the sites of photosynthetic reactions and contain the chlorophyll (created with BioRender.com). Scale bar: 10 μm.

After demonstrating the ability of **gPEI-Chi** to efficiently increase the CO_2_ that reacts with RuBisCO *in vitro*, we investigated the localization of the nanoparticles in the leaf tissue to gain insight on their potential for reaching the chloroplasts and increasing the CO_2_ amount at the photosynthetic sites *in vivo*. According to the lipid exchange envelope penetration (LEEP) model, infiltrated nanoparticles can spontaneously localize in the chloroplast depending on their size and surface charge. Nanoparticles must be within a specific range of size and zeta potential, to penetrate the negatively charged membrane of the chloroplasts and localize within their stroma.^58^ We quantified the ζ-potential of the **gPEI-Chi** nanoparticles in the aqueous infiltration buffer (pH 5.6) and found a small shift from 35.8 mV before CO_2_ bubbling to 37.4 mV after. The size and ζ-potential distributions, and possibility of aggregation of the nanoparticles, at the pH used for infiltration, indicate that some of the nanoparticles may passively enter the chloroplasts according to the LEEP model (Figure S17). We infiltrated CO_2_ loaded **gPEI-Chi** (**gPEI-Chi-CO**_**2**_) in tobacco leaves and, using confocal microscopy, we observed that the nanoparticles mainly localized in the leaf apoplast but could also penetrate the mesophyll cells in the infiltrated area and reach the chloroplasts (Figure 4). The colocalization of the **gPEI-Chi-CO**_**2**_ nanoparticles and chloroplasts was confirmed by recording the emission spectra of the selected regions (Figure 4A-iv,B-iv) and by calculating the Pearson and Mander’s colocalization coefficients (Figure S18). Furthermore, we could observe that the fluorescence of **gPEI-Chi-CO**_**2**_ followed the patterns of the discs stacks (grana) formed by the thylakoids inside the chloroplasts, which are the sites of photosynthetic reactions containing the chlorophyll which appear as the fluorescent red dots in the microscopy images (Figure 4C-iv). The confocal imaging confirms that the CO_2_-loaded nanoparticles can reach the photosynthetic sites and thus have the potential to increase the local CO_2_ concentration at the RuBisCO site and enhance the carboxylation reaction. In addition, we performed transmission electron microscopy (TEM) imaging of chloroplasts from plants infiltrated with **gPEI-Chi-CO**_**2**_ and found indications of the internalization of the nanoparticles in the chloroplasts, especially in the starch grains and around the stromal thylakoids (Figure S19).

**Figure 5:**
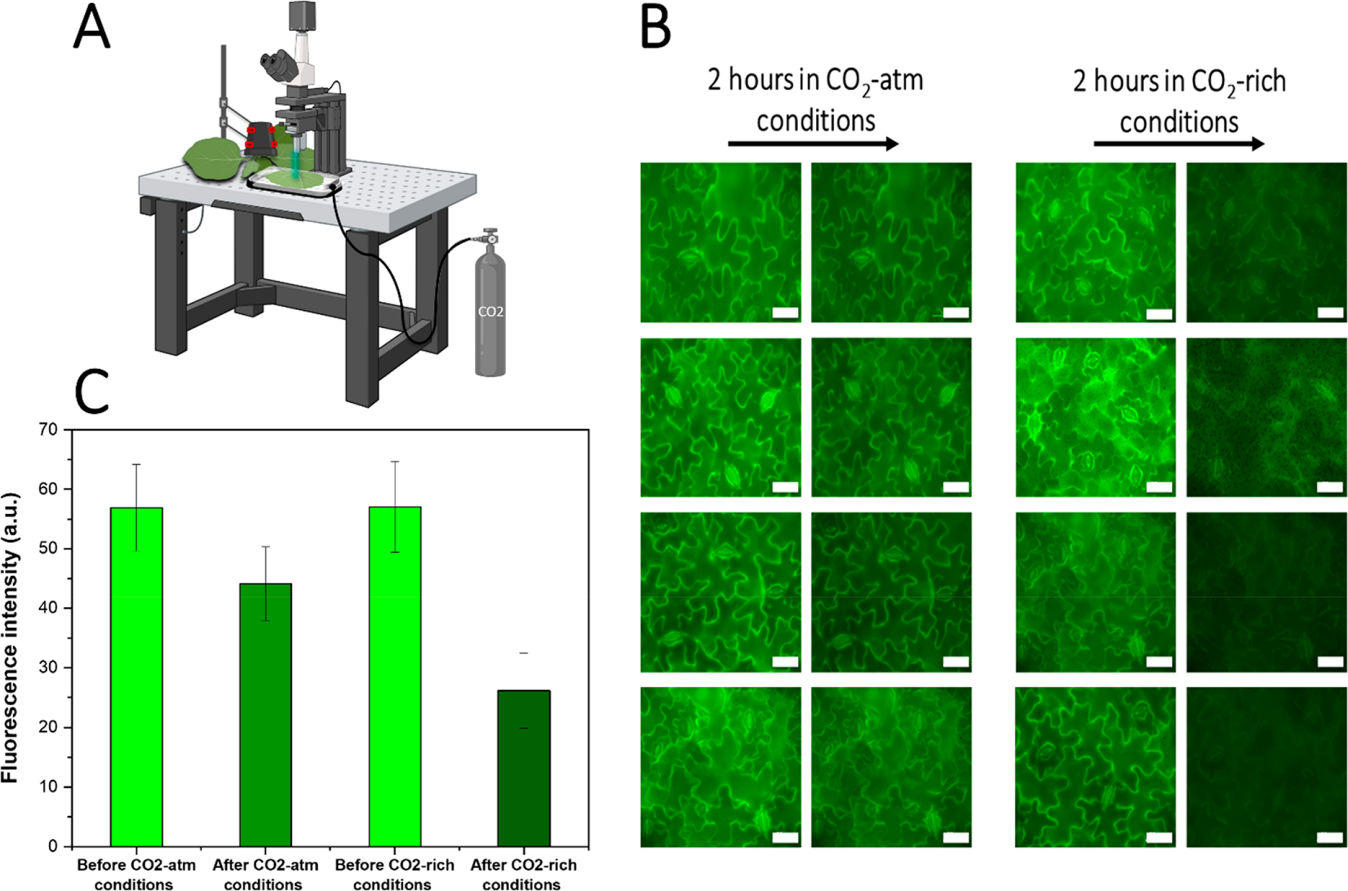
*In vivo* CO_2_ uptake of gPEI-Chi. (A) Scheme of the experimental setup where a leaf, still attached to its plant, is isolated in a CO_2_ incubator and imaged with fluorescence microscopy (created with BioRender.com). (B) Fluorescence microscopy of *Nicotiana tabacum* (tobacco) leaves infiltrated with **gPEI-Chi**, with or without incubation in a CO_2_-rich environment for 2 h, (*n* = 4 plants in each condition). Scale bar: 50 μm. (C) Fluorescence intensity evolution under atmospheric CO_2_ conditions or in a CO_2_-rich environment. Bars indicate the standard errors (*n* = 4 plants).

We then assessed whether the unloaded nanoparticles can uptake CO_2_ after infiltration to the plant as this will enable the reloading of the nanoparticles with atmospheric CO_2_ and the formation of a sustainable CO_2_-concentrating mechanism in planta. We first infiltrated both N_2_-bubbled and CO_2_-loaded **gPEI-Chi** in the apoplast of tobacco leaves (Figure S20) and confirmed that **gPEI-Chi** fluorescence maintained its CO_2_ dependence in the *in vivo* environment, i.e., **gPEI-Chi** fluorescence decreases when it is CO_2_-loaded. Next, to study the capacity of the nanoparticles to capture atmospheric CO_2_ while in the leaves, we infiltrated **gPEI-Chi** previously bubbled with N_2_ and then monitored the leaves fluorescence over time. The plants were imaged 1 day after infiltration to let any excessive infiltrated solvent to evaporate. The infiltrated leaves, still attached to the plant, were placed in a CO_2_-rich environment under a fluorescent microscope (Figure 5A) and the change in fluorescence intensity was evaluated after 2 h. As a control, we imaged infiltrated leaves that remained in atmospheric conditions. Figure 5B and C show that the fluorescence intensity decreased 54% for the leaves exposed to high CO_2_ concentration in comparison to decrease of 22% for the leaves let in atmospheric conditions, indicating that the nanoparticles in the apoplast of the plant are able to capture CO_2_. The small decrease in intensity in the control sample could also indicate that the nanoparticles can capture some CO_2_ when placed in atmospheric conditions, however, there is also the possibility of photobleaching of the dye after repetitive measurements at the same location. Furthermore, we measured the assimilation rate of infiltrated leaves at saturating light (A: the rate of CO_2_ uptake per unit time per unit leaf area (μmol of CO_2_ m^–2^ s^–1^)) and at different CO_2_ levels using the LI-6800 portable photosynthesis system and found that the infiltrated areas show higher CO_2_ uptake than controls 2 days after the infiltration (Figure S21). While these results are promising, more studies beyond the scope of this work are required to prove that the nanoparticles enhance plants photosynthetic activity *in vivo*.

## Conclusions

In conclusion, we designed chitosan-modified polyethyleneimine nanoparticles, **gPEI-Chi**, which can capture atmospheric CO_2_ in the form of bicarbonate and increase the CO_2_ that reacts with RuBisCO enzyme enhancing the carboxylation reaction by 20% in *in vitro* assay. The **gPEI-Chi** nanoparticles were infiltrated in tobacco leaves and, even after an extended period of time, they did not cause any adverse effect to the plant as PEI’s toxicity was obviated by the conjugated oligochitosan moieties. The CO_2_-loaded nanoparticles localized within the extracellular space, but most importantly, they could also pass cellular barriers and localize within the chloroplast organelles, which are the sites of photosynthetic activity. Using the **gPEI-Chi** CO_2_-dependent fluorescence, we also demonstrated the nanoparticles’ ability to uptake CO_2_*in vivo* when the leaf is placed in a CO_2_-rich environment while, in future studies, the fluorescence modulation can be extended to monitor internal CO_2_ concentrations. Overall, our results show that **gPEI-Chi** is a promising nanobiotechnological approach for integrating a CO_2_-concentrating mechanism in plants. CO_2_-loaded nanoparticles could potentially increase the CO_2_ concentration in the vicinity of the RuBisCO and then be reloaded by atmospheric CO_2_ when in planta. Furthermore, plants possess the carbonic anhydrase enzyme that converts bicarbonate to CO_2_, which can potentially increase the contribution of the **gPEI-Chi-**loaded nanoparticles since the conversion of bicarbonate to CO_2_ will be accelerated.^59,60^ However, to demonstrate the nanoparticles potential in planta, it will require their extended distribution throughout the plant’s photosynthetic sites. Although leaf infiltration is commonly used for introducing materials to plant leaves, the materials are usually distributed close to the infiltration site. Using vacuum infiltration in a detached leaf, we observed an extended distribution of the nanoparticles in the whole leaf area (Figure S22). Still, this method cannot be applied to intact plants apart from small seedlings that can fit into a vacuum chamber.^61,62^ Therefore, to evaluate the effect of the **gPEI-Chi** nanoparticles on RuBisCO activity and photosynthetic efficiency *in vivo*, another strategy for extended distribution of the nanoparticles to the plant photosynthetic sites has to be developed such as uptake via root or foliage.

## Experimental Section

### Synthesis of Oligochitosan

For this, 3.0 g of chitosan (MW = 15 kDa, deacetylation degree = 85%, Polysciences) was added to 150 mL of ultrapure water. Then 1.8 mL of HCl 37% was added and the mixture was stirred for 1 h until complete dissolution of the material. Afterward, 800 mg of NaNO_2_ was added and immediate N_2_ gas evolution was observed. After 16 h, the pH was neutralized with AmberLite IRN78 OH. After filtration, the addition of acetone (300 mL) and centrifugation (8000 rpm, 5 min) allowed the recovery of the chitosan oligomers (oligochitosan). The material was redispersed in a minimum amount of water, precipitated again by acetone addition, and collected by centrifugation. Finally, it was washed with methanol and with acetone. After vacuum-drying, 1.8 g of oligochitosan in the form of a white powder was obtained.

### Synthesis of **PEI-Chi**

In a round-bottom flask, 200 mg of branched polyethyleneimine (MW = 2 kDa, Polysciences) was dissolved in 30 mL of 1.5% acetic acid solution in water (v/v); then 600 mg of oligochitosan was added and the stirring solution was heated up to 40 °C. Three separate additions of sodium cyanoborohydride of 150 mg each were performed after 1 h, 6 h, and 24 h. After 3 days, the solution was poured in a larger vessel, 1 mL of triethylamine was added first, and then, after 10 min stirring, 60 mL of acetone, achieving the precipitation of **PEI-Chi**. The solid was recuperated by centrifugation (8000 rpm, 5 min); thus, it was redispersed in a minimum amount of water, precipitated again by acetone addition, and collected by centrifugation. Finally, it was washed with methanol and with acetone. After vacuum-drying, 540 mg of **PEI-Chi** in the form of a beige powder was obtained.

### Functionalization of **PEI-Chi** with Fluorescein 5-isothiocyanate

In a round-bottom flask, 130 mg of **PEI-Chi** was dispersed in 50 mL of water, followed by the addition of 0.3 mL of triethylamine. A second solution was prepared by dissolving 5 mg of fluorescein 5-isothiocyanate in 10 mL of dimethyl sulfoxide. Then the fluorescein solution was added slowly to the **PEI-Chi** solution while stirring. After 1 day, **gPEI-Chi** was precipitated by the addition of acetone. The product was collected by centrifugation and washed with a mixture of acetone/ethanol 2:1, where free fluorescein is highly soluble. When no more fluorescein was detected in the supernatant, the precipitate was washed with acetone only and, after vacuum-drying, 96 mg of **gPEI-Chi** in the form of an orange powder was obtained.

### NMR Spectra

NMR spectra were recorded at 298 K with a 400.33 MHz Bruker Advance spectrometer. Deuterated solvents and NMR tubes were purchased from Eurisotop. Branched polyethyleneimine (MW 2 kDa) and Chitosan (MW 15 kDa, deacetylation degree 85%) were purchased from Polysciences. Sodium cyanoborohydride (95%), sodium nitrite (97%), triethylamine (99.5%), acetic acid (glacial, 99%) were purchased from Sigma-Aldrich. Fluorescein 5-Isothiocyanate (isomer I, 97%) was purchased from TCI.

### Size Exclusion Chromatography

Acetic acid aqueous solution was used as the mobile phase at a flow rate of 1.00 mL/min and glycerol as a flow rate marker. All polymers were injected (100 μL of solution) at a concentration of 5 mg/mL after filtration through a 0.45 μm pore-size membrane. The separation was carried out on two Agilent columns [2 × PLgel 5 μm Mixed C (300 × 7.5 mm)] and a guard column (PL gel 5 μm). Columns and detectors were maintained at 40 °C. Relative Mw was determined thanks to a conventional calibration obtained with polyethylene glycol narrow standards.

### UV–vis/NIR Absorption Spectra

UV–vis/NIR absorption spectra were obtained using an Agilent Cary 5000 instrument. Spectra were recorded between 200 and 800 nm, using a 10 mm side quartz cuvette. The absorption spectra were measured both before and after CO_2_ loading of the nanoparticles. The loading was carried out by gently bubbling carbon dioxide through a needle plunged in the polymer water solutions for 5 min.

### Steady-State Fluorescence

Steady-state fluorescence measurements were carried out with a Horiba FluoroMax 4 spectrofluorimeter using a 10 mm side quartz cuvette. The excitation and emission spectra were measured both before and after CO_2_ loading of the nanoparticles The loading was carried out by gently bubbling carbon dioxide through a needle plunged in the polymer water solutions for 5 min.

### Fourier Transform Infrared Spectroscopy

Fourier transform infrared spectroscopy (FTIR) measurements were performed on a Nicolet iS 5N FTIR spectrometer. Spectra were collected from 4000 to 400 cm^–1^ with the following settings: 16 scans per sample, and spectral resolution: 4 cm^–1^.

### Atomic Force Microscopy

Atomic force microscopy (AFM) (Dimension Fast Scan, Bruker) was used in a tapping mode using a silicon cantilever (Fastscan-A) with a nominal tip radius of 5 nm. The samples were prepared by drop-casting solution of polymer (0.1 mg/mL, 3000 pm, 120 s) on a mica substrate, which was subsequently dried at room temperature for 1 day and on a hot plate at 60 °C for 5 min.

### ζ-Potential Measurements

ζ-potential measurements were determined using a Zetasizer (NanoZS90, Malvern, United Kingdom). First, nanoparticles were suspended in morpholinoethanesulfonic acid (MES, Sigma-Aldrich) (pH 5.6 adjusted with KOH) and the nanoparticle surface charge was determined using zeta potential measurement (averaged over 10 measurements each one having 20 runs).

### Plant Material and Growth Condition

*Nicotiana tabacum SR1* (tobacco) seeds were sown in the soil and grown in a growth chamber (Percival, CLF PlantClimatics GmBH, Wertingen, Germany) at 120 μmol m^–2^ s^–1^ light intensity, 60% relative humidity, and a 12/12 h day/night regime at 24 °C during the day and 18 °C during night-time. All experiments and measurements were performed using 6 weeks old plants.

### Infiltration of *Nicatiana tabacum* (Tobacco)

Plants were illuminated with light of high intensity of ∼500 μmol m^–2^ s^–1^ for 1 h to make sure the stomata are open. Then, a small hole was made on the abaxial side of the tobacco leaf with the tip of a sterile 0.8 × 50 mm HENKE-JECT syringe needle (1 hole per leaf infiltrated). It is important to note that the hole does not completely puncture through the leaf but only passes the epidermal layer in order to facilitate the infiltration of the solution in the plant tissue. Once the hole was made, 200–300 μL of a 0.1 mg/mL **gPEI-Chi** solution in 10 mM MES, 10 mM MgCl_2_, pH 5.6 (or just 10 mM MES, 10 mM MgCl_2_, pH 5.6 as control) was infiltrated from the hole with a 1 mL needleless syringe. In order to control the applied pressure and not to destroy the plant tissue, the researcher put their finger on the adaxial side of the leaf to maintain in place the syringe on the abaxial side of the leaf and then by gently pushing the plunger of the syringe to infiltrate the solution in the leaf. Any solution administered on the surface of the leaf was rinsed with water and dried gently with a piece of clean tissue. For microscopy experiments, the infiltrated plants were let to stand 24 to 48 h postinfiltration in normal growth conditions to allow remaining solvent to evaporate, while for toxicity experiments and staining they were let to stand in normal growth conditions up to 1 to 2 weeks.

### Propidium Iodide Staining

Propidium iodide staining was performed by cutting, with a clean razor blade, the leaf of interest and dipping in for 20 min in a 20 μmol aqueous solution of propidium iodide (Sigma-Aldrich, St. Louis, MO, USA) in a 50 m conical tube (Sarstedt, Nümbrecht, Germany). The leaf was then removed from the solution and excess staining was removed by rinsing in water. The stained leaves were then directly used for microscopy.

### Fluorescence Microscopy

Widefield fluorescence imaging was performed with a Ni-E Upright Motorized Microscope (Nikon) equipped with a Zyla sCMOS camera (Andor Technology). The source of illumination was a Lambda DG-4 ultrahigh speed wavelength switching illumination system (Sutter Instrument). The Propidium Iodide emission was detected between 580 and 650 nm using a TRITC filter, a 50*x*/0.60 TU PLAN ELWD objective and an exposure time of 1s. The **gPEI-Chi** emission was detected between 510 and 560 nm using a FITC filter, a 50*x*/0.60 TU PLAN ELWD objective and an exposure time of 200 ms. The images were treated using the software NIS-Elements (Nikon) and ImageJ. The samples used for microscopy were prepared by cutting a small leaf section of the infiltrated or stained leaf tissue and inserting it between a glass slide (VWR international GmbH, Darmstadt, Germany) and Menzel-Gläzer coverslip (VWR international GmbH, Darmstadt, Germany) using a 30% glycerol aqueous solution as mounting medium to keep the sample moisturized during imaging.

### RuBisCO Assays

The enzymatic assays for determining the activity of RuBisCO were realized following the method described by *Yasumoto et al.*.^37^ The chemicals were purchased from Sigma-Aldrich (St. Louis, MO, USA).

### 3-Phosphoglyceric Acid Determination by LC-MS

3-Phosphoglyceric acid determination by LC-MS was performed at the Swedish Metabolomics Center in Umeå, Sweden. Before the analysis, 200 μL of freeze-dried sample was resuspended in 50 + 50 μL methanol and water. The chromatographic separation was performed on an Agilent 1290 Infinity UHPLC-system (Agilent Technologies, Waldbronn, Germany). One μL of each sample were injected onto an Atlantis Premier BEH-HILIC, 1.7 μm, 2.1 × 50 mm^2^, held at 40 °C. The gradient elution buffers were composed of (A) 10 mmol/L ammonium acetate in water of pH 9 and (B) 10 mmol/L ammonium acetate in 10/90 water/acetonitrile (v/v) of pH 9 (pH of aqueous buffer before mixing with methanol). Ammonium hydroxide was used to adjust pH of the mobile phase. A final 5 μM concentration of methylenediphosphonic acid was spiked into the solvents for the analysis. The flow rate was 0.35 mL/min, and the compounds were eluted with a linear gradient consisting of 95–30% B over 7 min, B was held at 30% for 2 min; B was increased to 95% for 0.5 min and held for 1.5 min, then the flow-rate was increased to 0.6 mL min^–1^ for 1.5 min; these conditions were held for 0.2 min, after which the flow-rate was reduced to 0.35 mL min^–1^ for 0.1 min before the next injection.

The compounds were detected with an Agilent 6546 Q-TOF mass spectrometer equipped with a jet stream electrospray ion source operating in negative ion mode. A reference interface was connected for accurate mass measurements; the reference ions purine (4 μM) and HP-0921 (Hexakis(1H, 1H, 3H-tetrafluoropropoxy)phosphazine) (1 μM) were infused directly into the MS at a flow rate of 0.05 mL min^–1^ for internal calibration, and the monitored ions were purine *m*/*z* 119.03632; HP-0921 *m*/*z* 966.000725. The gas temperature was set to 150 °C, the drying gas flow to 8 L min^–1^ and the nebulizer pressure 35 psig. The sheath gas temp was set to 350 °C and the sheath gas flow 11 L min^–1^. The capillary voltage was set to 4000 V. The nozzle voltage was 300 V. The fragmentor voltage was 120 V, the skimmer 65 V and the OCT 1 RF Vpp 750 V. The collision energy was set to 0 V. The *m*/*z* range was 70–1700, and data was collected in centroid mode with an acquisition rate of 4 scans s^–1^ (1977 transients/spectrum). Data were processed and analyzed using MassHunter Qualitative Analysis, Quantitative Analysis (QqQ; Agilent) and Excel (Microsoft) software.

Methanol, HPLC-grade, was obtained from Fischer Scientific (Waltham, MA, USA) Acetonitrile, HPLC-grade was obtained from Fischer Scientific (Waltham, MA, USA) 2-Propanol, HPLC-grade was obtained from VWR (Radnor, PA, USA) H_2_O, Milli-Q, Ammonia solution, 35% HPLC-grade was obtained from Fischer Scientific (Waltham, MA, USA). Reference and tuning standards: Purine, 4 μM, Agilent Technologies (Santa Clara, CA, USA) HP-0921 (Hexakis(1H, 1H, 3H-tetrafluoropropoxy)phosphazine), 1 μM, Agilent Technologies (Santa Clara, CA, USA) Calibrant, ESI-TOF, ESI-L Low Concentration Tuning Mix, Agilent Technologies (Santa Clara, CA, USA) HP-0321 (Hexamethoxyphosphazine), 0.1 mM, Agilent Technologies (Santa Clara, CA, USA).

### Confocal Microscopy Imaging

Confocal imaging was performed with an inverted Zeiss LSM 980 (Carl Zeiss AG, Oberkochen, Germany) confocal microscope equipped with an Airyscan2 detection unit. The objective Plan-Apochromat 63*x*/1.4 Oil DIC M27 (FWD = 0.19 mm, Carl Zeiss AG, Oberkochen, Germany) was used, with Immersol 518 F immersion media (*n* = 1.518, Carl Zeiss AG, Oberkochen, Germany) and all images were processed with the Zen Blue software 3.4.

We evaluated the contribution of the polymer at each wavelength using the LSM lambda scan spectrophotometer, and compared images of the green and red channels by using both the confocal and the Airyscan2 super-resolution modes. We captured images in 16 bits with a bidirectional acquisition, an averaging of 4 images, an image size of 1863 × 1863 pixels (pixel size = 0.043 μm) and a zoom of 1.3. Both the fluoresceine isocyanate (FITC) and chlorophyll were excited using a 488 nm laser at an intensity of 0.4% with a pinhole of 5 AU, a detector gain at 700 V and a pixel time of 1.13 μs. The Alexa Fluorophore 520 (emission wavelength 520 nm) emission filter was used for the detection of **gPEI-Chi**, and the Alexa Fluorophore 700 (emission wavelength 719 nm) emission filter was used for the detection of the chlorophyll. The samples used were prepared by cutting a small leaf section of tobacco tissue infiltrated with **gPEI-Chi** preliminary loaded with CO_2_ (**gPEI-Chi-CO**_**2**_) and inserting it between a glass slide (VWR international GmbH, Darmstadt, Germany) and Menzel-Gläzer coverslip (VWR international GmbH, Darmstadt, Germany) using a 30% glycerol aqueous solution as mounting medium to keep the sample moisturized during imaging. We gently removed via capillarity the extra mounting medium between the glass slide and the coverslip in order to create a suction effect that allows to turn the samples upside down for imaging with the inverted microscope.

### CO_2_ Incubation

Twenty-four to forty-eight hours postinfiltration with **gPEI-Chi**, the plants were fixed upside down on the fluorescent microscope platform and the infiltrated leaf was isolated in a closed Petri dish having an entry tube for CO_2_ and a small hole allowing air circulation. The fluorescence of **gPEI-Chi** was first imaged in atmospheric conditions; then we filled the Petri dish with CO_2_ (coming from a CO_2_ tank) for 2 h and captured the fluorescence again.

## Supporting Information Available

Figures giving additional details on material investigation including NMR, HSQC, IR spectroscopy, AFM, and SEC; confocal microscopy images investigating long-term toxicity, detectability, and colocalization with chloroplasts *in vivo.*; transmission electron microscopy of infiltrated samples; emission spectra and fluorescence microscopy to study CO_2_-dependent fluorescence of nanoparticles; assimilation-CO_2_ response curves of infiltrated plants (PDF)
