# Supplementary material for: Chitosan-Modified Polyethyleneimine Nanoparticles for Enhancing the Carboxylation Reaction and Plants’ CO2 Uptake
Source: ACS Nano. 2023 Feb 16;17(4):3430–41. doi: 10.1021/acsnano.2c09255 (PMC9979637; doi:10.1021/acsnano.2c09255)
Supplement: Supplementary file 1 — nn2c09255_si_001.pdf [file nn2c09255_si_001.pdf]

Supporting information for:

Chitosan-modified polyethyleneimine nanoparticles for enhancing the carboxylation reaction and plants' CO_2 uptake

Cyril Routier[1^], Lorenzo Vallan[2^], Yohann Daguerre[3], Marta Juvany[3], Emin Istif[2], Daniele Mantione[2,4], Cyril Brochon[2], Georges Hadziioannou[2], Åsa Strand[5], Torgny Näsholm[3], Eric Cloutet[2], Eleni Pavlopoulou[2,6], Eleni Stavrinidou[1,3]*

[1] Laboratory of Organic Electronics, Department of Science and Technology, Linköping University, SE-60174, Norrköping, Sweden.
[2] Laboratoire de Chimie des Polymères Organiques (LCPO−UMR 5629), Université de Bordeaux, Bordeaux INP, CNRS, F-33607 Pessac, France.
[3] Umeå Plant Science Centre, Department of Forest Genetics and Plant Physiology, Swedish University of Agricultural Sciences, SE-90183 Umeå, Sweden.
[4] POLYMAT, University of the Basque Country UPV/EHU, 20018 San Sebastián, Spain
[5] Umeå Plant Science Centre, Department of Plant Physiology, Umeå University, SE 901-87 Umeå, Sweden.
[6] Institute of Electronic Structure and Laser, Foundation for Research and Technology—Hellas, P.O. Box 1527, 71110 Heraklion Crete, Greece.

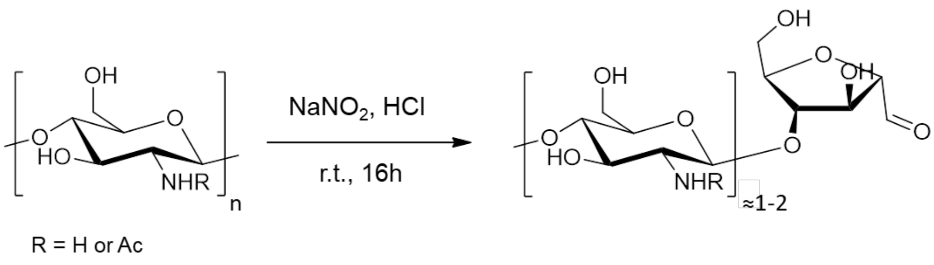

Figure S1. Chemical route for the synthesis of oligochitosans.

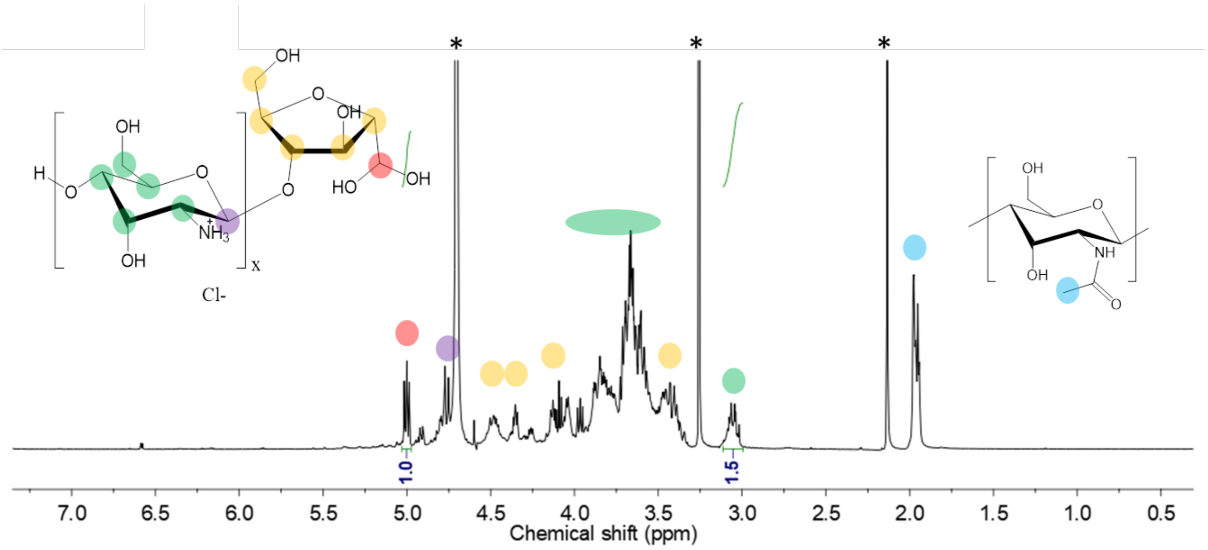

Figure S2. ^{1}H NMR of oligochitosan in D_2O with 1% DCl. Signal attribution is corroborated by the work of *A. Moussa et al.*.[1] The aldehyde proton is found at 5.01 ppm, meaning that the aldehyde is in the hydrated form due to the acidic environment. The integral ratio between the signal at 3.06 ppm and the aldehyde signal at 5.01 ppm is 1.5, indicating that, in average, each 2,5-anhydro-D-mannofuranose (amf) reducing end is connected to one or two 2-amino-2-deoxy-β-D-glucopyranose (GlcN) units and/or 2-acetamido-2-deoxy-β-D-glucopyranose (GlcNAc) units. Solvents peaks (*) belong to water (4.70 ppm), MeOH (3.26 ppm) and acetone (2.13 ppm).

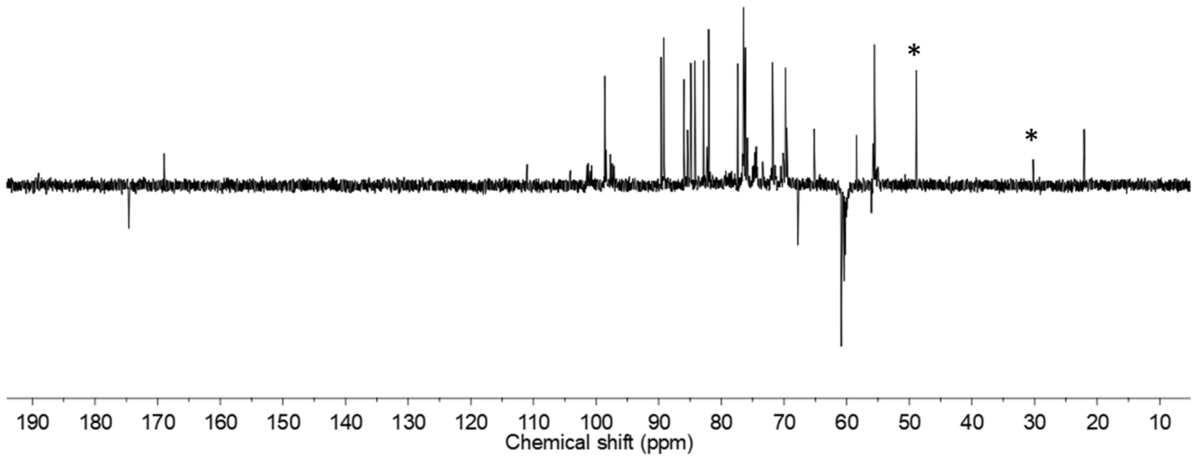

Figure S3. APT NMR of oligochitosan in D_2O with 1% DCl. Positive phase is assigned to primary and tertiary carbons. Peaks at 101 and 97 ppm are related to hydrated aldehydes with similar chemical environment. As the peak at 169 ppm suggests, the acidic environment promotes the reversible formation of imine between the amine and the aldehydes of oligochitosans. Solvents peaks (*) belong to MeOH (49 ppm) and acetone (30 ppm).

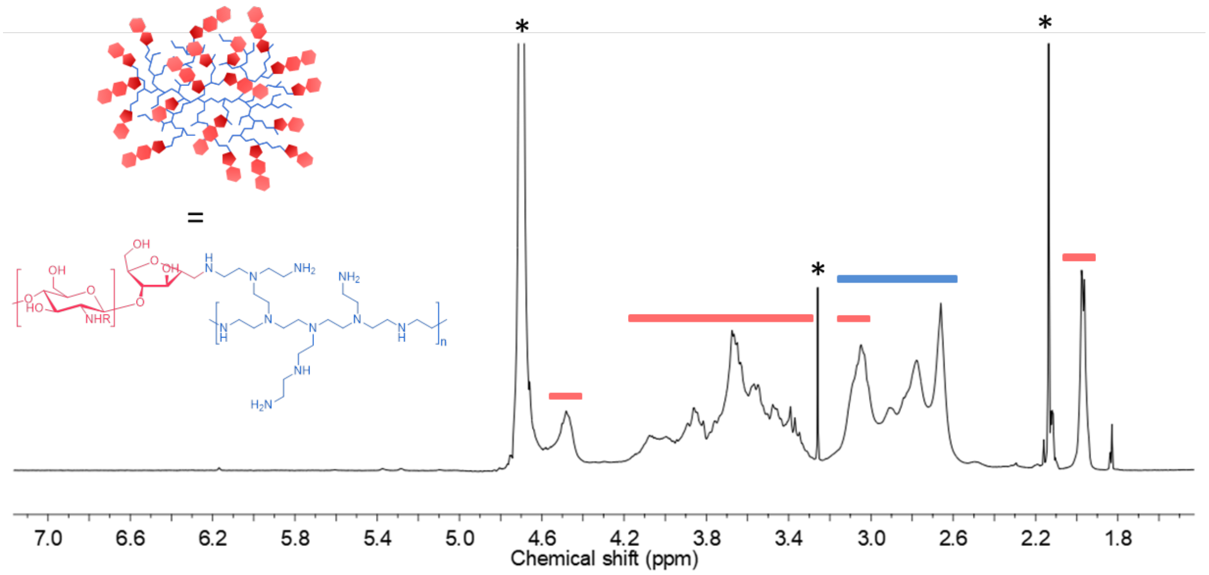

Figure S4. ^{1}H NMR of **PEI-Chi** in D_2O with 1% DCl. The effectiveness of the reductive amination is proved by the absence of the aldehyde signal at 5.01 ppm. The signals at 4.45 ppm, between 4.1 and 3.3 ppm and at 1.96 ppm belong to the oligochitosan structure, while the signals between 3.15 and 2.60 ppm belongs to the methylenes of the branched polyethyleneimine structure. Solvents peaks (*) belong to water (4.70 ppm), MeOH (3.26 ppm) and acetone (2.13 ppm).

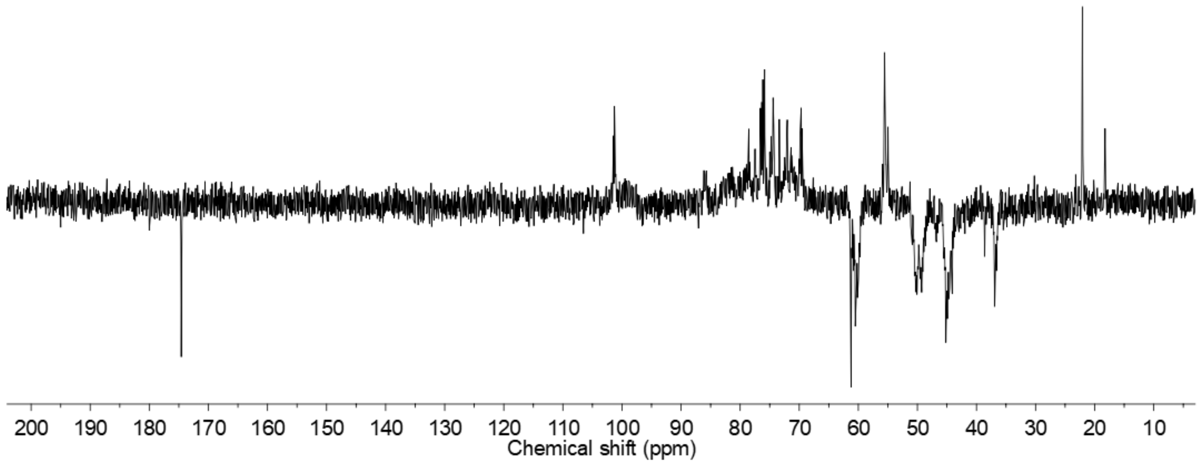

Figure S5. APT NMR of **PEI-Chi** in D_2O with 1% DCl. Positive phase is assigned to primary and tertiary carbons. The methylene carbons of branched polyethyleneimine appear in the negative phase at 50-49, 45-44 and 37 ppm.

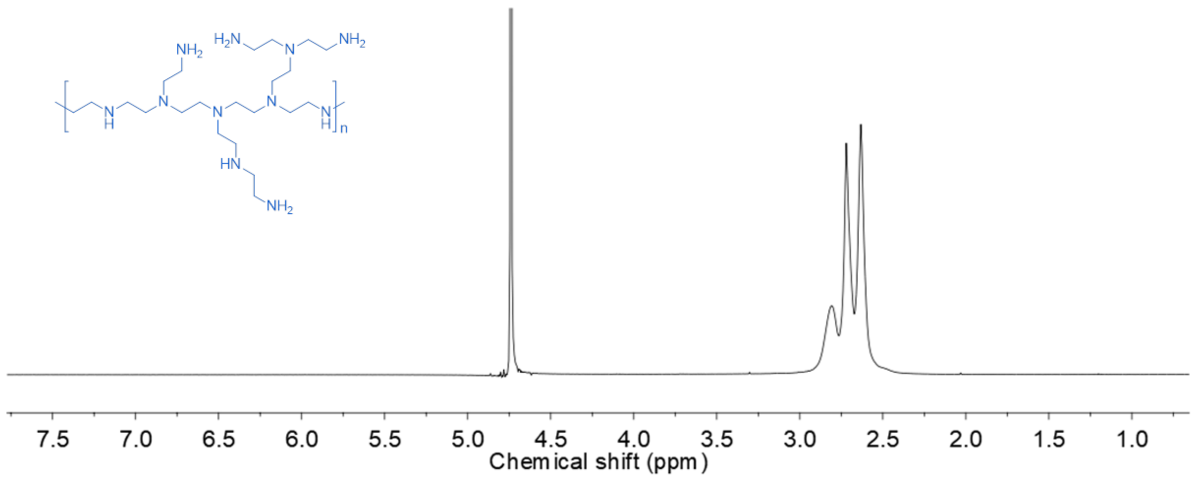

Figure S6. ^{1}H NMR of **PEI 2kDa** in D_2O with 1% DCl.

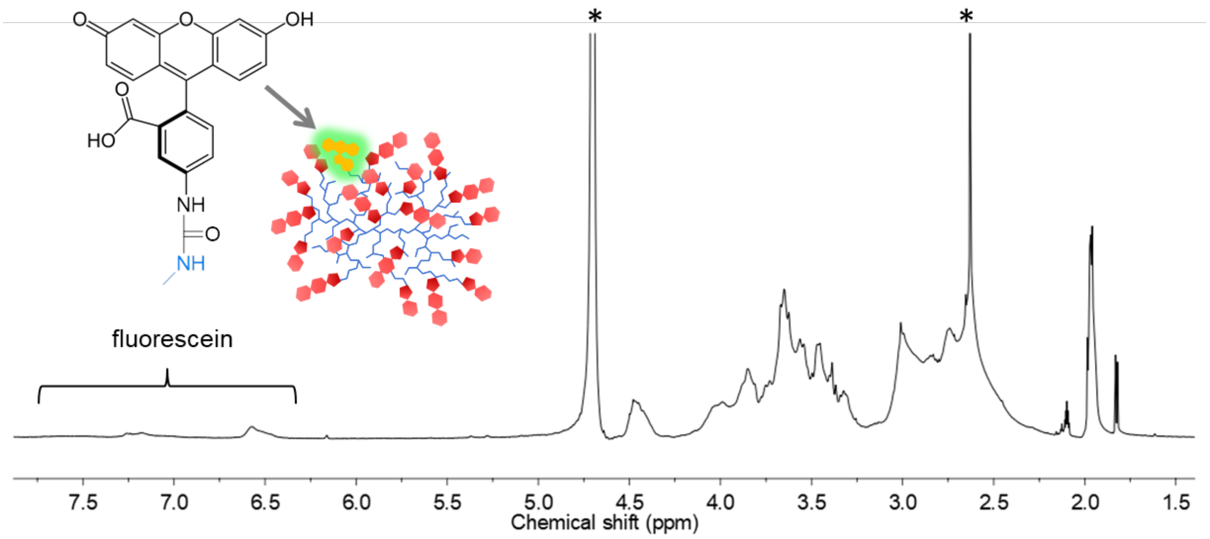

Figure S7. ^{1}H NMR of **gPEI-Chi** in D_2O with 1% DCl. Weak signals at 7.6, 7.2 and 6.6 ppm are related to fluorescein protons. Solvents peaks (*) belong to water (4.70 ppm) and DMSO (2.63 ppm).

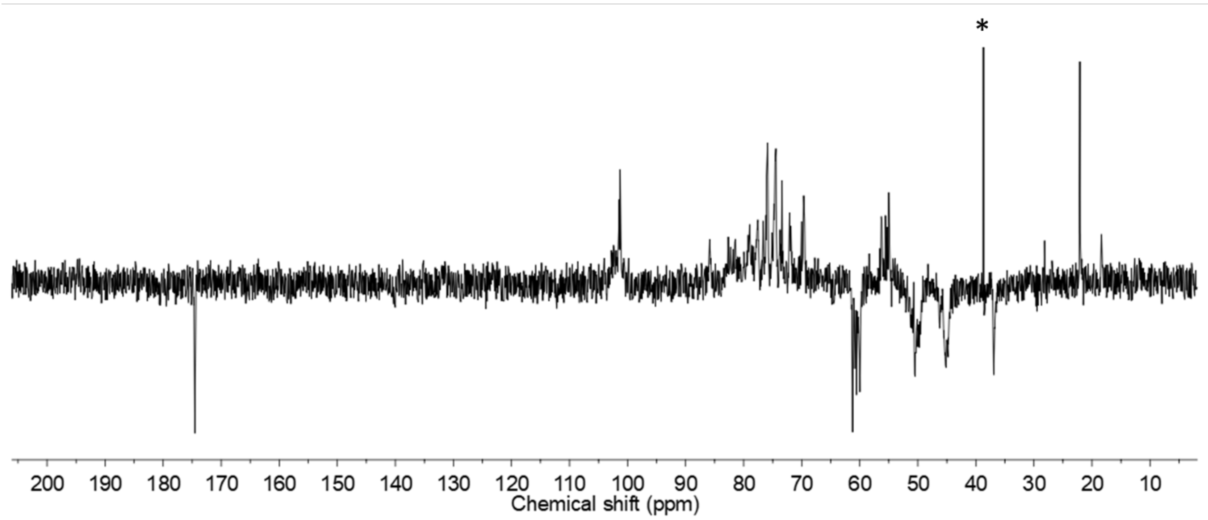

Figure S8. APT NMR of **gPEI-Chi** in D_2O with 1% DCl. Positive phase is assigned to primary and tertiary carbons. Solvents peak (*) belongs to DMSO (39 ppm).

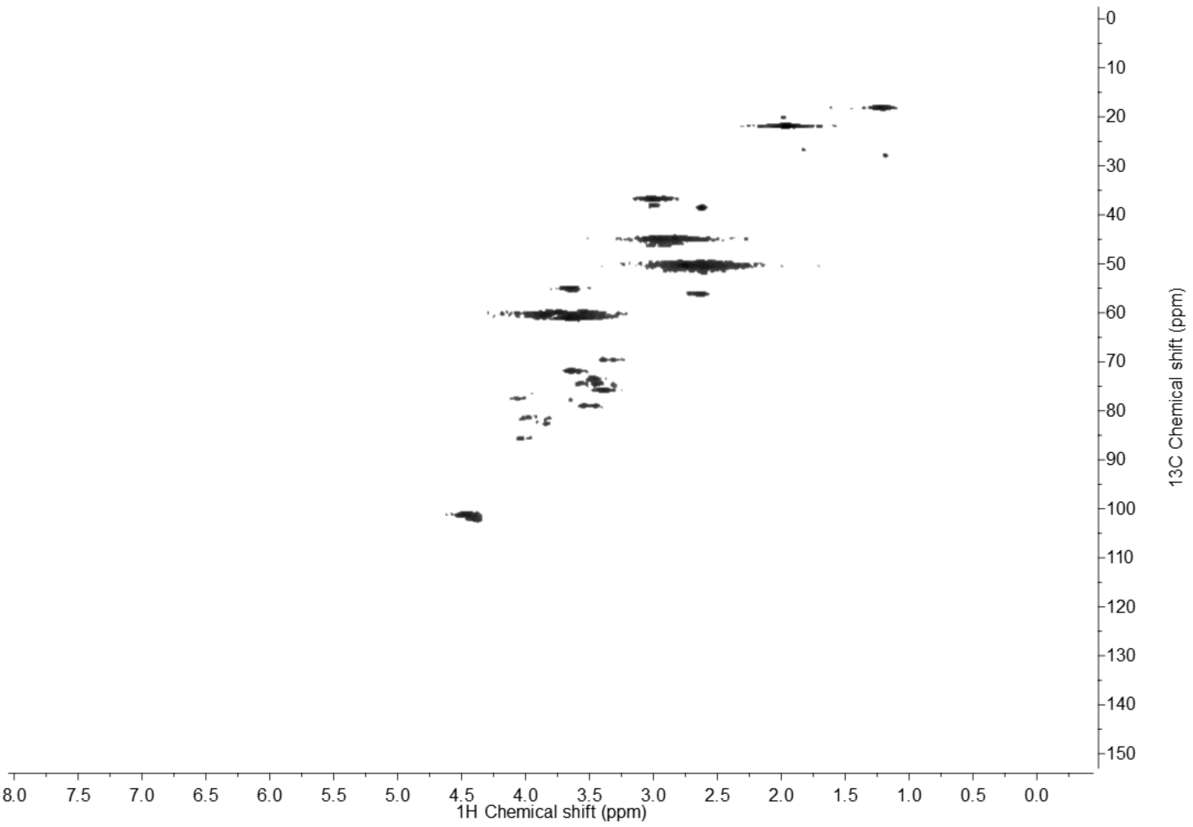

Figure S9. HSQC spectrum of **gPEI-Chi** in D_2O with 1% DCl.

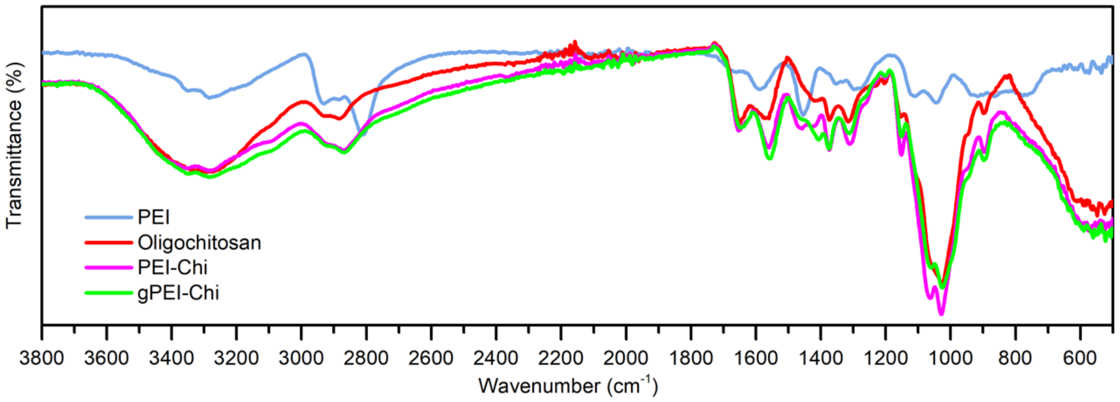

Figure S10. IR spectra of **PEI 2 kDa**, **oligochitosan**, **PEI-Chi** and **gPEI-Chi**.

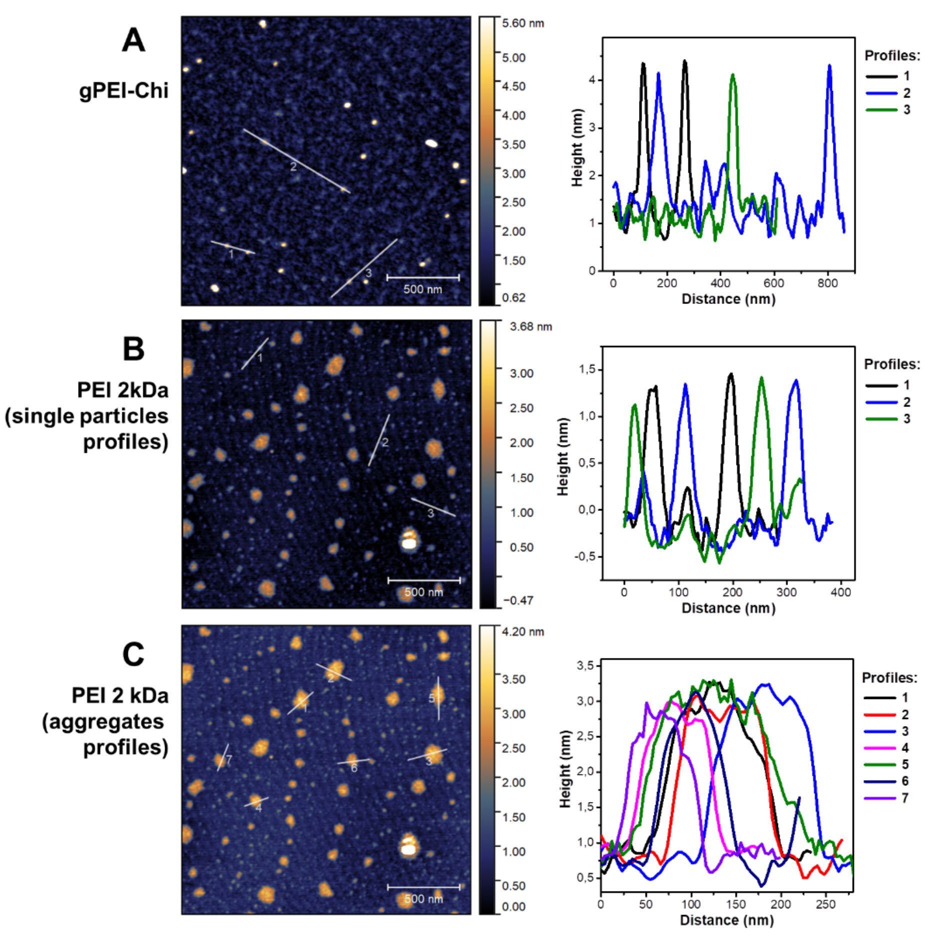

Figure S11. (**A**) AFM image and height profiles of **gPEI-Chi**, (**B**) AFM image and height profiles of the individual PEI nanoparticles and (**C**) of PEI aggregates.

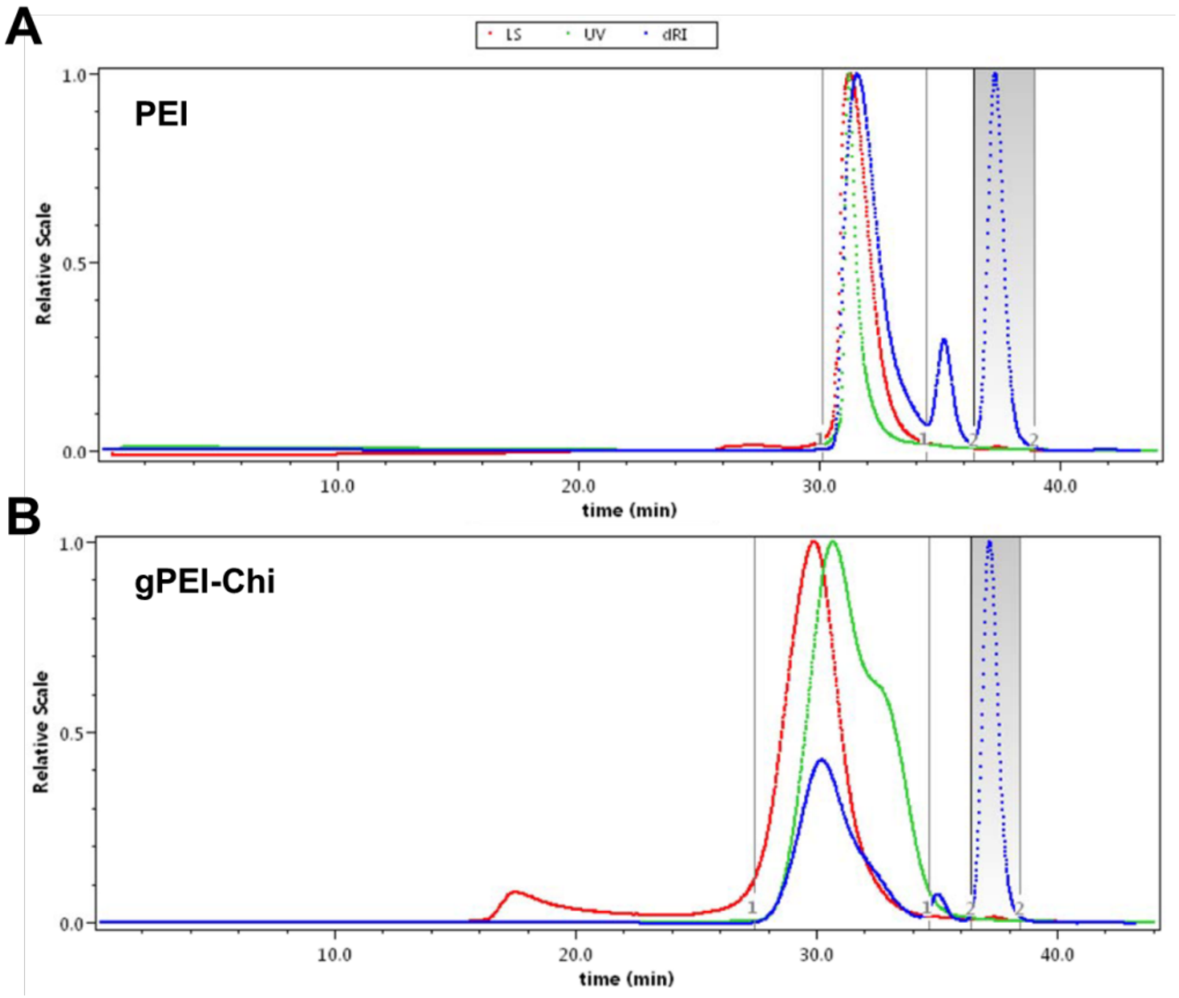

Figure S12. SEC analysis by light scattering (red), UV (green) and refractive index (blue) of (**A**) **PEI** and (**B**) **gPEI-Chi**.

Table S13. Mn, Mw, Mz and dispersity of PEI and gPEI-Chi estimated by SEC.

	Mn	Mw	Mz	Dispersity (Mw/Mn)
PEI	0.95 kDa	1.24 kDa	1.44 kDa	1.3
gPEI-Chi	1.62 kDa	2.89 kDa	3.41 kDa	1.62

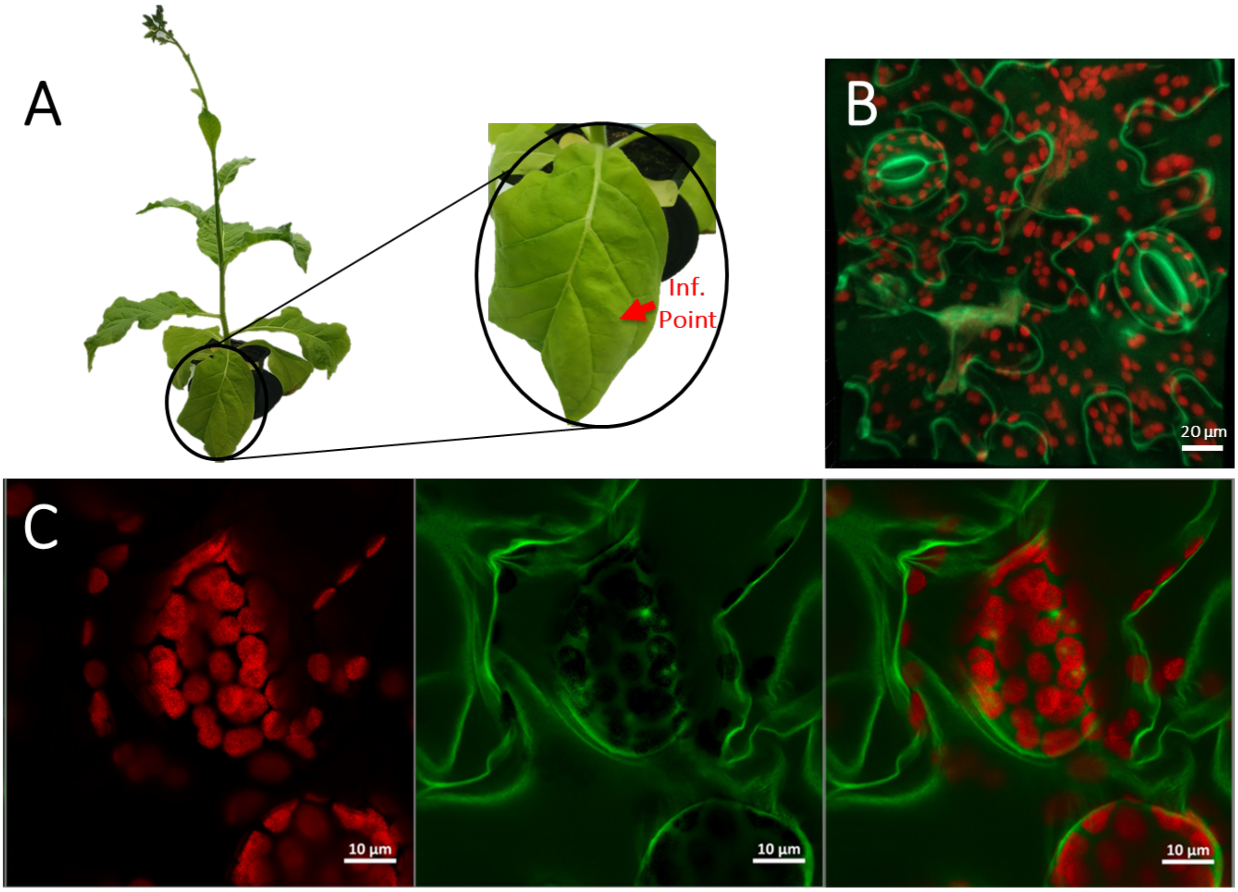

Figure S14. Tobacco plant shows no signs of toxicity one month after **gPEI-Chi** nanoparticles infiltration and the fluorescence of the nanoparticles is still detectable. No fluorescence was detected beyond the infiltrated area indicating that the polymer did not diffuse through the plant after the infiltration. (**A**) Tobacco plant one month after infiltration with zoom in image on the infiltrated leaf area. (**B**) Confocal microscopy Z-stack image of the leaf infiltrated with **gPEI-Chi** one month after infiltration. Scale bar: 20 µm. (**C**) Airyscan confocal super-resolution microscopy of a leaf infiltrated with **gPEI-Chi** one month after infiltration. The left red panel shows the autofluorescence of the chlorophyll, the middle green panel shows the **gPEI-Chi** fluorescence, and the right panel is a merged image of the two channels. Scale bar: 10 µm.

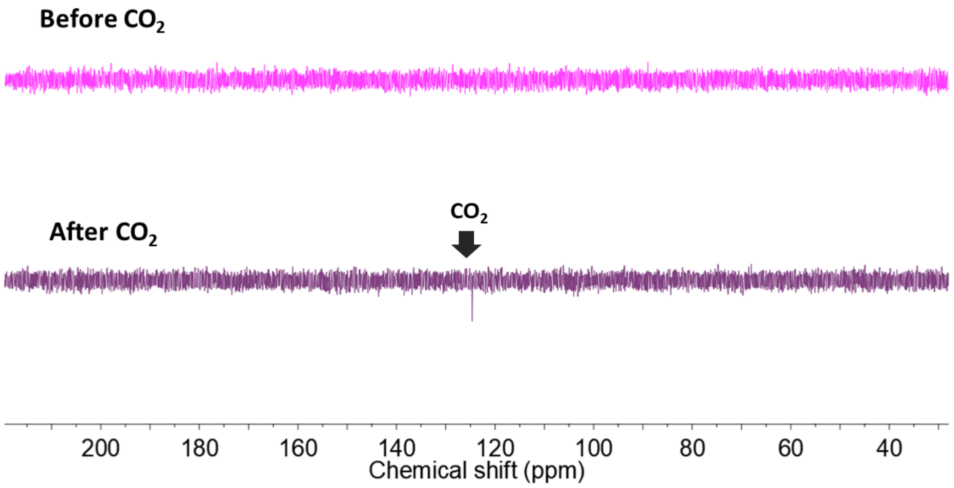

Figure S15: APT NMR spectra of **PEI-Chi** in D_2O before and after bubbling carbon dioxide.

To prove that CO_2 loading in solution is correlated with the decrease in fluorescence we measured the fluorescence spectra of solutions with different CO_2 loading concentrations. We made two **gPEI-Chi** solutions; one that is saturated in CO_2 (by bubbling CO_2 for one hour) obtaining **gPEI-Chi-CO$_2$**, and one that is CO_2 free (by bubbling N_2 for 2 days in solution) obtaining **gPEI-Chi-N$_2$**. We then measured the fluorescent spectra using a microplate reader for different mixing concentrations of those two solutions, hence obtaining spectra for different levels of CO_2 in solution. Furthermore, to mimic the infiltration solution, we used the infiltration buffer as the solvent and not pure water as in **Figure 2D**. The fact that we observe the change in fluorescence even in the presence of a buffer further validates our hypothesis that small local changes in pH due to CO_2 binding with **PEI-Chi** will induce a change in the nanoparticles' fluorescence and thus **gPEI-Chi** may be used *in vivo* to monitor CO_2 dynamics.

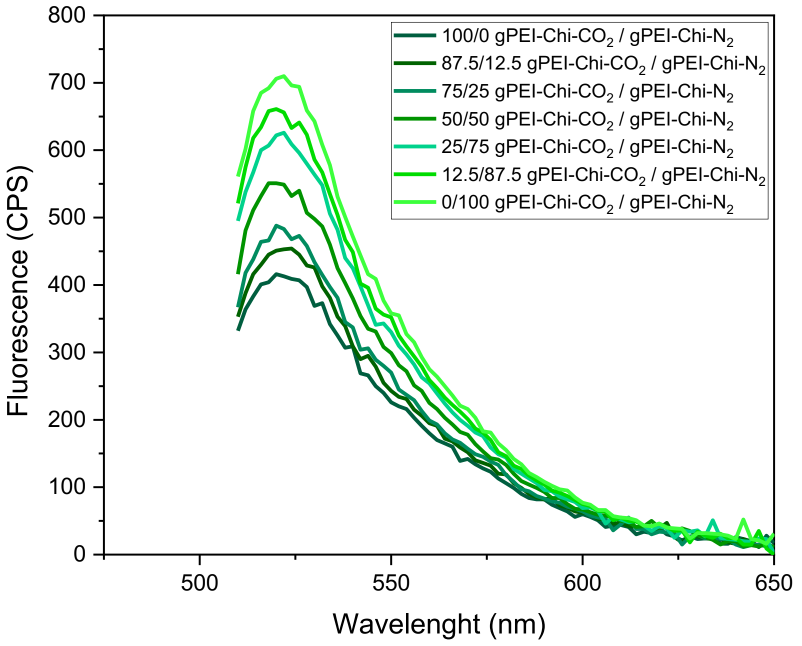

Figure S16. Emission spectra of **gPEI-Chi** (0.001 mg/ml) in 10 mM (MES) (pH 5.6) and 10 mM $MgCl_2$ for different level of CO_2 saturation in solution. Spectra acquired with microplate reader BioTek Synergy H1 between 510-700 nm with 2 nm step and with excitation wavelength λexc=480 nm.

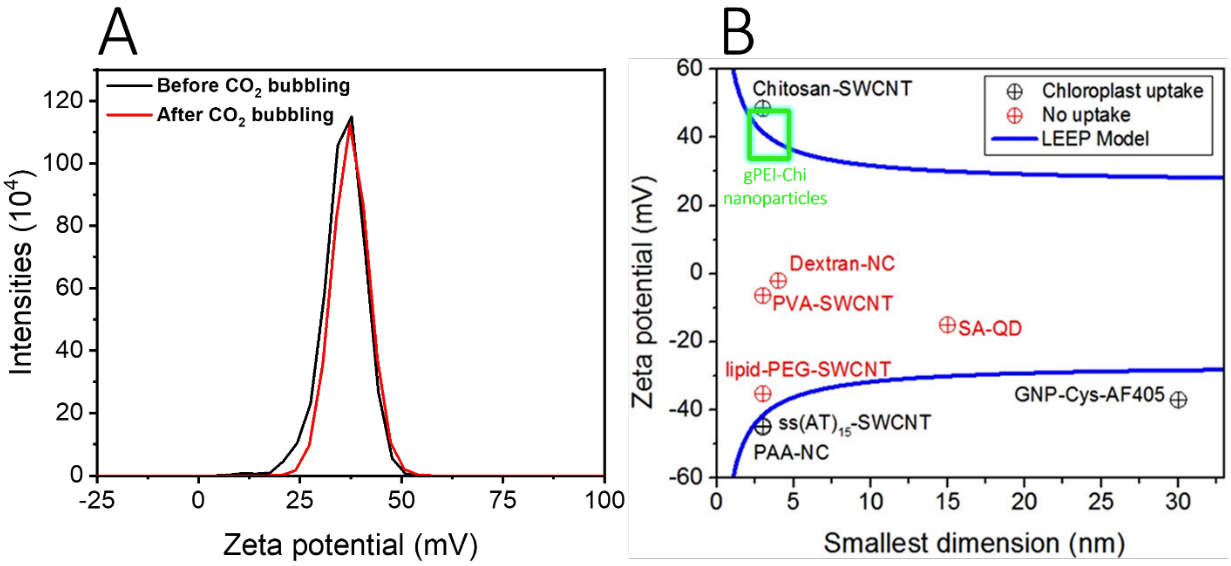

Figure S17. (**A**) ζ-potential distributions of **gPEI-Chi** before (black) and after (red) bubbling CO_2 in solution. (**B**) Figure extracted and adapted from the paper published by *Wong, M. H. et al.*[2]. The green square represents where the **gPEI-Chi** stands according to their size and ζ-potential distributions.

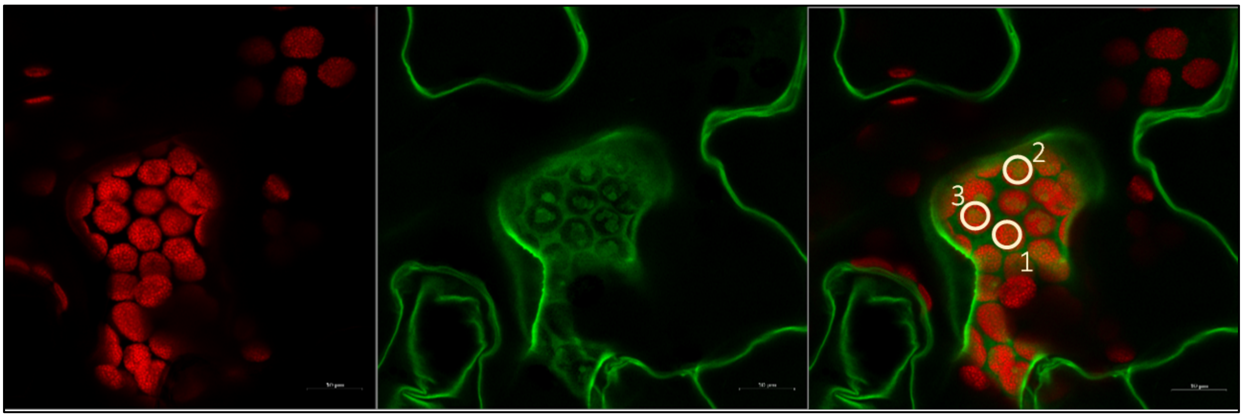

Position	Manders Coefficient	Pearson
1	0.94	-0.64
2	0.93	-0.71
3	0.93	-0.53

Figure S18. Manders and Pearsons coefficients for colocalization between the **gPEI-Chi** and the chlorophyll.

We also analyzed by Transmission electron microscopy (TEM) pristine tobacco leaves (control 1), samples infiltrated with the buffer solution only (control 2) and samples infiltrated with **gPEI-Chi-CO_2**, in order to see if the loaded nanoparticles can locate in the chloroplasts.

Experimental Procedure for TEM Microscopy: One day after infiltration (to let the solvent evaporate), 2x2 mm samples from the leaves were fixed in 2,5% GA (glutaraldehyde) in 0,1 M PB (phosphate buffer pH 7,2-7,4). When kept in the fixative solution, the samples can be kept for several weeks before analysis but one day in the fixative medium is enough.

Before microscopy, the samples were rinsed with a phosphate buffer 3 times for 10 min, incubated with 1% OsO_4 in the dark for two hours, rinsed with Milli-Q water 3 times for 10 min, dehydrated in ethanol gradients for 15 min (25%, 50%, 75%, 90%, 95% and 100%) and the last dehydration step was repeated in 100% ethanol. Then, the samples were infiltrated in different gradients of resin and 100% ethanol (1:3, 1:1 and 3:1) for one hour in each step. Samples were left in 1:1 step overnight in the fume hood. Next, the samples were incubated in 100% resin for an hour, then the resin was replaced by a fresh one, left in the fume hood for an hour and polymerised in the oven at 60°C overnight.

The samples were then trimmed with a razor blade and sectioned with a diamond knife to produce 70 nm sections that were picked up on a 100 mesh EM grid. The sections were then post-stained with 5% uranyl acetate and lead citrate and imaged on a Talos L120C TEM microscope with a Ceta 16M CCD camera.

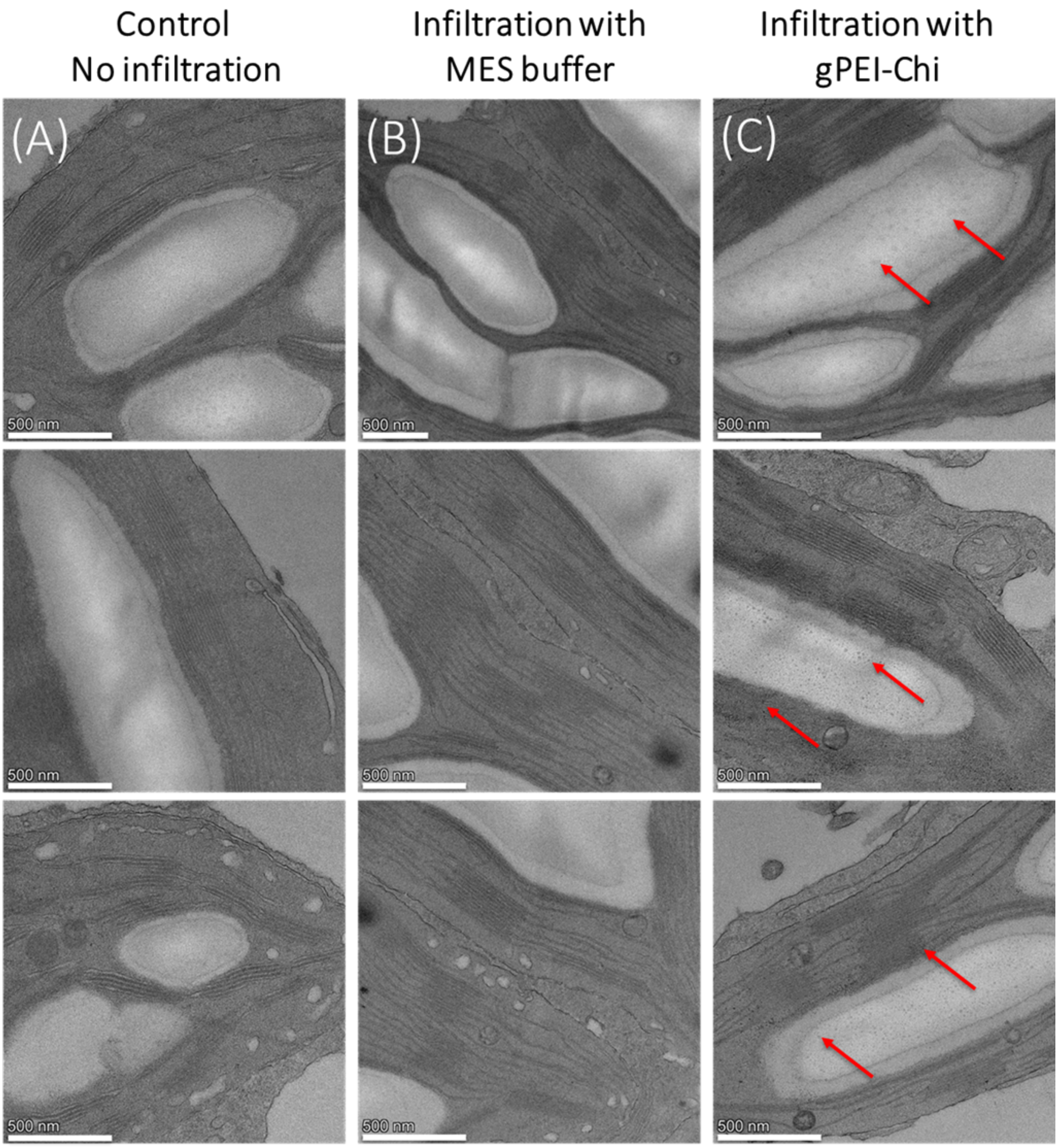

Figure S19. Transmission electron microscopy (TEM) images of chloroplasts from (**Column A**) pristine tobacco leaves, (**Column B**) leaves infiltrated with a MES buffer solution, (**Column C**) leaves infiltrated with **gPEI-Chi-CO_2**. Scale bar: 500 nm. Red arrows possibly show the NPs as these structures are observed only in the treated samples.

In comparison with the controls, we observed that the samples infiltrated with **gPEI-Chi-CO_2** nanoparticles presented darker spots especially in the starch grains of the chloroplasts and grainier structures around the stromal thylakoids that could be due to the presence of the nanoparticles. The method employed however also presented black spots in several samples

(controls and **gPEI-Chi**) which could be due to the lead used in the staining process and would be considered as artifacts. While these results are encouraging more studies are required with higher magnification and without the use of lead.

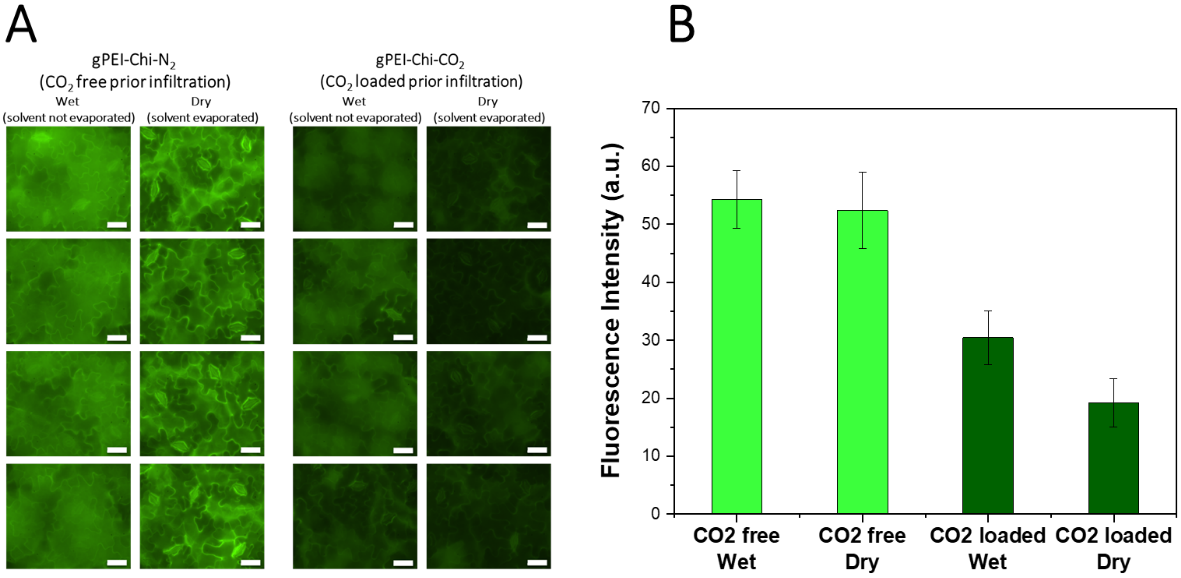

Figure S20. Fluorescence intensity depending on the preloading of **gPEI-Chi** with CO_2 prior infiltration. (**A**) Fluorescence microscopy of a *Nicotiana Tabacum* (tobacco) leaf infiltrated with **gPEI-Chi**, with or without CO_2 preloading before infiltration. Scale bar: 50 µm. (**B**) Fluorescence intensity. Bars indicate the standard errors (n = 4 plants).

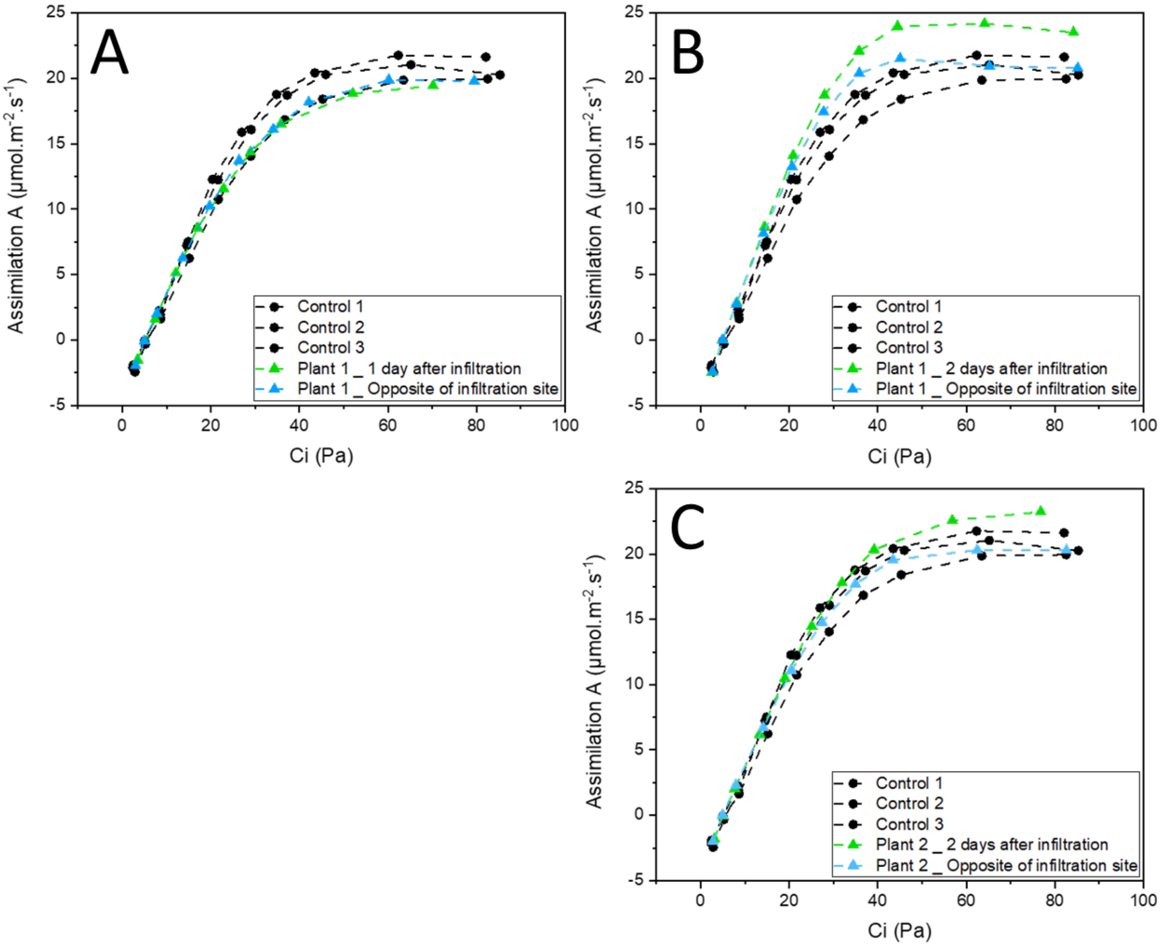

Figure S21. Assimilation-CO_2 response curves (A-Ci) using *Nicotiana Tabacum* (tobacco) plants exposed to a range of CO_2 concentrations (10, 50, 100, 200, 300, 400, 500, 600, 800, 1000 ppm) at saturating light (thus not limiting photochemistry). In black are three control plants without infiltration. In green and blue are plants that are infiltrated with gPEI-Chi-CO_2; for the green curve, the measurement was made on the site of infiltration, while for the blue curve, the measurement was made on the opposite side of the infiltrated leaf where no infiltration was made. (A), (B) correspond to measurements performed on the same plant, 1 and 2 days after infiltration respectively. (C) corresponds to a second plant where measurements were performed 2 days after infiltration. The curves were measured with the LI-6800 Portable Photosynthesis System- Porometer/Fluorometer (LI-COR).

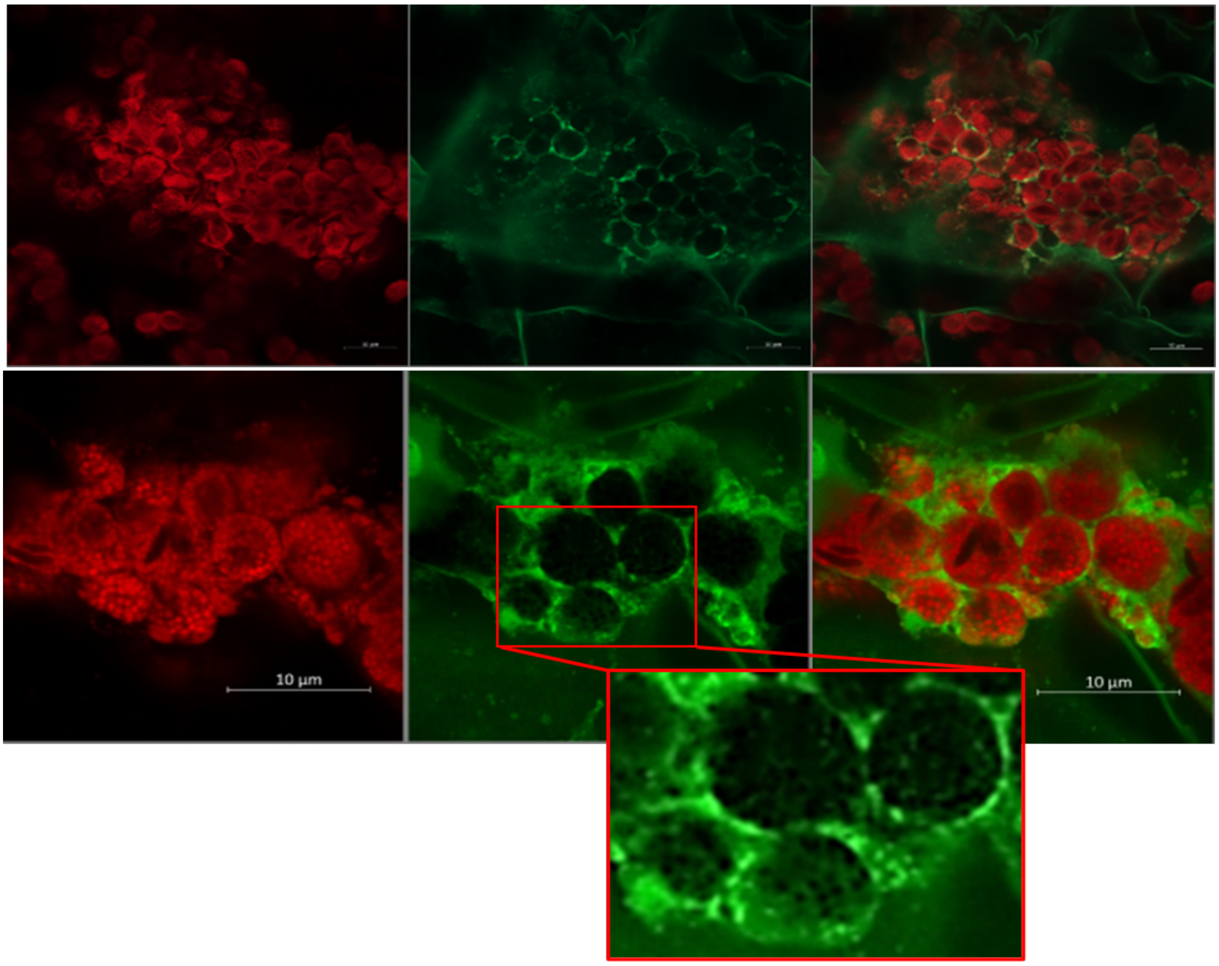

Figure S22. Vacuum infiltration of **gPEI-Chi** in tobacco.

References:

1. Moussa, A.; Crépet, A.; Ladavière, C.; Trombotto, S. Reducing-End "Clickable" Functionalizations of Chitosan Oligomers for the Synthesis of Chitosan-Based Diblock Copolymers. *Carbohydr. Polym.* **2019**, *219*, 387–394.
2. Wong, M. H.; Misra, R. P.; Giraldo, J. P.; Kwak, S. Y.; Son, Y.; Landry, M. P.; Swan, J. W.; Blankschtein, D.; Strano, M. S. Lipid Exchange Envelope Penetration (LEEP) of Nanoparticles for Plant Engineering: A Universal Localization Mechanism. *Nano Lett.* **2016**, *16*, 1161–1172.
